# Towards artificial intelligence-based learning health system for population-level mortality prediction using electrocardiograms

**DOI:** 10.1038/s41746-023-00765-3

**Published:** 2023-02-06

**Authors:** Weijie Sun, Sunil Vasu Kalmady, Nariman Sepehrvand, Amir Salimi, Yousef Nademi, Kevin Bainey, Justin A. Ezekowitz, Russell Greiner, Abram Hindle, Finlay A. McAlister, Roopinder K. Sandhu, Padma Kaul

**Affiliations:** 1 Department of Computing Science, University of Alberta, Edmonton, AB Canada; 2 Canadian VIGOUR Centre, Department of Medicine, University of Alberta, Edmonton, AB Canada; 3 Alberta Machine Intelligence Institute, University of Alberta, Edmonton, AB Canada; 4 Department of Medicine, University of Alberta, Edmonton, AB Canada; 5 Smidt Heart Institute, Cedars-Sinai Medical Center Hospital System, Los Angeles, CA USA

**Keywords:** Outcomes research, Prognosis

## Abstract

The feasibility and value of linking electrocardiogram (ECG) data to longitudinal population-level administrative health data to facilitate the development of a learning healthcare system has not been fully explored. We developed ECG-based machine learning models to predict risk of mortality among patients presenting to an emergency department or hospital for any reason. Using the 12-lead ECG traces and measurements from 1,605,268 ECGs from 748,773 healthcare episodes of 244,077 patients (2007–2020) in Alberta, Canada, we developed and validated ResNet-based Deep Learning (DL) and gradient boosting-based XGBoost (XGB) models to predict 30-day, 1-year, and 5-year mortality. The models for 30-day, 1-year, and 5-year mortality were trained on 146,173, 141,072, and 111,020 patients and evaluated on 97,144, 89,379, and 55,650 patients, respectively. In the evaluation cohort, 7.6%, 17.3%, and 32.9% patients died by 30-days, 1-year, and 5-years, respectively. ResNet models based on ECG traces alone had good-to-excellent performance with area under receiver operating characteristic curve (AUROC) of 0.843 (95% CI: 0.838–0.848), 0.812 (0.808–0.816), and 0.798 (0.792–0.803) for 30-day, 1-year and 5-year prediction, respectively; and were superior to XGB models based on ECG measurements with AUROC of 0.782 (0.776–0.789), 0.784 (0.780–0.788), and 0.746 (0.740–0.751). This study demonstrates the validity of ECG-based DL mortality prediction models at the population-level that can be leveraged for prognostication at point of care.

## Introduction

Developing a learning health system, one that follows a cycle of routinely collecting and analysing health data to generate new knowledge that can be applied to inform health decisions or system improvements, is a major priority for Canada and other countries^1^. Canada’s publicly-funded, universal health care system has allowed for the deterministic linkage of health care data from different healthcare settings (hospitals, emergency departments, outpatient clinics, and physician offices) to insurance and vital statistics registries to identify predictors of mortality and develop risk stratification algorithms^2–4^. These models have been enhanced by the availability of pharmaceutical claims and laboratory data at the population-level in some provinces^5–8^.

The electrocardiogram (ECG) is a readily available, low-cost diagnostic tool performed on a majority of patients during an acute care visit and contains important information about the structure and electrical activity of the heart^9^. In recent years, exponential advances in computational resources and machine learning technologies, coupled with digitized ECG datasets have opened up opportunities for ECG-based diagnostic and prognostic predictions^10–12^. However, the feasibility and value of linking ECG data to longitudinal population-level administrative health data to assist clinicians at point of care decision-making with the goal of completing the cycle of quality and facilitating a learning healthcare system has not been previously explored^1,13^.

This motivated us to use a large population-level cohort of patients with universal health insurance presenting to emergency departments or hospitals to develop ECG-based machine learning models to predict both short-term (30-day) and longer-term mortality (1- and 5-year). We explored the added utility of incorporating laboratory (lab) values available at the time of the ECG to the models’ prediction performance, and examined the performance of the models in specific sex and diagnostic subgroups.

## Results

### Patient characteristics and outcomes

Characteristics of patient cohorts used in the study are described in Table 1. The average age of patients at the time of the ECG in both the development and holdout sets was 65.8 years. Recall, however, that we randomly selected one ECG per patient from the holdout set to use for the final evaluations. The average age in this latter set was slightly lower at 62.6 years. This is likely because older patients had more ECGs than younger ones. Similarly, men had more ECGs, so the proportion of men was slightly lower in the random evaluation set than in the development or holdout sets (54.7% vs 56.7%). This pattern was observed for some of the ECG measurements (e.g., mean of QRS duration: 97.9 vs 101.3 ms; QT interval 395.1 vs 399.8 ms), comorbidities (e.g., heart failure: 4.1% vs 6.2%; atrial fibrillation 9.2% vs 15.5%) as well as lab values (e.g., troponin I 2.0 vs 2.2 µg/L; creatinine 107.5 vs 116.5 µmol/L, Table 2).

**Table 1:** Characteristics of patient cohorts used in the study.

	Full Data (*n* = 1,605,268)	Development set (*n* = 964,741)	Holdout set (*n* = 640,527)	Random ECG per patient in holdout set^a^ (*n* = 97,631)
Age (years)	65.80 ± 17.25	65.77 ± 17.22	65.85 ± 17.29	62.57 ± 18.59
Sex (Male in %)	56.73	56.81	56.6	54.73
ECG measurements				
Atrial rate	85.60 ± 46.15	85.56 ± 46.11	85.67 ± 46.20	84.06 ± 40.30
P duration	155.92 ± 116.60	156.00 ± 116.39	155.79 ± 116.91	163.96 ± 114.06
RR interval	790.81 ± 213.11	790.90 ± 212.63	790.68 ± 213.83	790.89 ± 204.35
Q wave onset	508.84 ± 6.51	508.82 ± 6.29	508.87 ± 6.82	509.04 ± 6.17
Fridericia Rate-Corrected QT interval	434.86 ± 38.05	434.96 ± 38.04	434.71 ± 38.07	429.55 ± 35.23
Heart Rate	81.64 ± 23.22	81.61 ± 23.18	81.69 ± 23.28	81.13 ± 21.94
PR interval	169.34 ± 38.46	169.44 ± 37.66	169.18 ± 39.65	165.99 ± 33.65
QRS duration	101.36 ± 24.26	101.40 ± 24.23	101.31 ± 24.30	97.89 ± 21.66
QT interval	399.81 ± 54.83	399.94 ± 54.74	399.63 ± 54.96	395.10 ± 51.41
Bazett’s Rate-Corrected QT interval	455.02 ± 40.09	455.10 ± 40.09	454.89 ± 40.09	449.23 ± 37.16
Frontal P axis	44.85 ± 35.52	44.81 ± 35.41	44.91 ± 35.69	45.66 ± 32.19
Frontal QRS axis in Initial 40 ms	27.50 ± 46.30	27.44 ± 46.37	27.59 ± 46.20	28.48 ± 42.12
Frontal QRS axis in Terminal 40 ms	45.36 ± 88.15	45.74 ± 88.28	44.80 ± 87.96	46.24 ± 84.64
Frontal QRS axis	19.98 ± 54.37	20.04 ± 54.38	19.88 ± 54.37	22.68 ± 49.81
Frontal ST wave axis	90.94 ± 88.23	90.98 ± 88.07	90.87 ± 88.48	79.23 ± 85.11
Frontal T axis	55.70 ± 67.76	55.48 ± 67.60	56.03 ± 68.00	47.97 ± 59.90
Horizontal P axis	20.69 ± 47.30	20.63 ± 47.14	20.77 ± 47.52	21.01 ± 41.15
Horizontal QRS axis in Initial 40 ms	27.79 ± 48.39	27.86 ± 48.17	27.69 ± 48.71	30.20 ± 42.68
Horizontal QRS axis in Terminal 40 ms	34.10 ± 129.50	33.94 ± 129.40	34.35 ± 129.66	26.67 ± 125.58
Horizontal QRS axis	−0.91 ± 78.19	−1.11 ± 77.91	−0.61 ± 78.62	−4.03 ± 69.27
Horizontal ST wave axis	97.02 ± 64.99	96.98 ± 65.00	97.09 ± 64.97	91.75 ± 60.20
Horizontal T axis	64.46 ± 58.98	64.27 ± 58.96	64.73 ± 59.01	59.30 ± 53.05
Comorbidities				
Peripheral Vascular Disease	33,518 (2.09%)	19,714 (2.04%)	13,804 (2.16%)	2144 (2.20%)
Cerebrovascular Disease	54,349 (3.39%)	33,191 (3.44%)	21,158 (3.30%)	4252 (4.36%)
Hypertension	350,859 (21.86%)	210,275 (21.80%)	140,584 (21.95%)	15,387 (15.76%)
Dementia	133,963 (8.35%)	80,037 (8.30%)	53,926 (8.42%)	8849 (9.06%)
Chronic Pulmonary Disease	31,764 (1.98%)	19,215 (1.99%)	12,549 (1.96%)	2078 (2.13%)
Diabetes Mellitus	120,260 (7.49%)	71,860 (7.45%)	48,400 (7.56%)	5684 (5.82%)
Renal Disease	163,262 (10.17%)	96,924 (10.05%)	66,338 (10.36%)	8800 (9.01%)
Liver Disease	20,268 (1.26%)	12,062 (1.25%)	8206 (1.28%)	1079 (1.11%)
Cancer	18,905 (1.18%)	11,707 (1.21%)	7198 (1.12%)	1346 (1.38%)
NSTEMI	93,946 (5.85%)	55,632 (5.77%)	38,314 (5.98%)	8699 (8.91%)
STEMI	162,274 (10.11%)	96,828 (10.04%)	65,446 (10.22%)	6534 (6.69%)
Heart Failure	100,206 (6.24%)	60,381 (6.26%)	39,825 (6.22%)	4049 (4.15%)
Atrial Fibrillation	249,325 (15.53%)	150,055 (15.55%)	99,270 (15.50%)	8958 (9.18%)

ECG measurements and Comorbidities are expressed in terms of the number of ECG instances.

^a^Descriptives are provided as mean ± standard deviation using one iteration of random ECG sampling in the holdout set. *ECG* electrocardiogram, *N* number, *NSTEMI* non-ST-elevation myocardial infarction, *STEMI* ST-elevation myocardial infarction.

**Table 2:** Lab characteristics of patient cohorts used in the study (as mean ± standard deviation).

	Full Data (*n* = 601,307)	Development set (*n* = 361,585)	Holdout set (*n* = 239,722)	Random ECG per patient in holdout set (*n* = 30,076)
Age (years)	64.82 ± 17.57	64.90 ± 17.53	64.70 ± 17.62	64.74 ± 17.87
Sex (Male in %)	55.84	55.81	55.88	56.63
Lab measurements				
GFR (mL/min)	66.01 ± 28.03	65.85 ± 28.04	66.25 ± 28.03	69.66 ± 27.94
Creatinine (umol/L)	116.46 ± 117.03	117.20 ± 119.62	115.35 ± 113.00	107.52 ± 100.63
Hemoglobin (g/L)	126.53 ± 23.23	126.46 ± 23.27	126.65 ± 23.18	127.81 ± 23.32
Potassium (mmol/L)	4.02 ± 0.64	4.02 ± 0.64	4.02 ± 0.63	3.99 ± 0.62
Sodium (mmol/L)	137.39 ± 4.35	137.38 ± 4.36	137.41 ± 4.33	137.39 ± 4.39
Troponin I (ug/L)	2.24 ± 10.07	2.23 ± 10.09	2.24 ± 10.04	1.99 ± 9.49

*ECG* electrocardiogram, *GFR* glomerular filtration rate, *N* number.

**Fig. 1:**
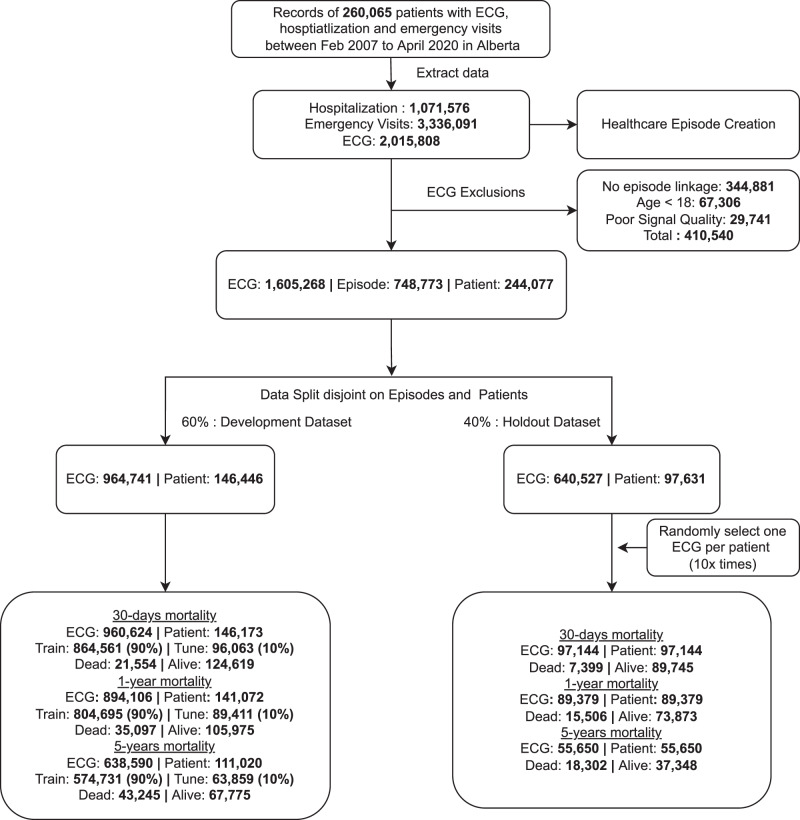
Study cohort, data splits and evaluation scheme. Flowchart of study design, showing sample sizes for overall study, experimental splits and different outcomes. We divided the overall ECG dataset into a random split of 60% for the model development (for training (90% subset of the 60%) and fine tuning (10% subset of the 60%) the model), and the remaining 40% as the holdout set for validation. Patients with more severe illnesses are expected to undergo ECGs more frequently, and more ECGs may be acquired at terminal stages when patients are monitored more regularly. To mitigate potential bias in model performance due to differential representation of patient phenotypes, we evaluated our models using a single randomly-selected ECG per patient from their multiple episodes in the holdout set.

The models for 30-days, 1-year, and 5-year mortality were trained on 146,173, 141,072, and 111,020 patients and evaluated on 97,144, 89,379, and 55,650 patients, respectively (Fig. 1). In our evaluation set of one random ECG per holdout patient, 7,399 (7.6%), 15,506 (17.3%), 18,302 (32.9%) had died at 30 days, 1 year, and 5 years, respectively. Similarly, for the subset of ECGs for which lab values were available, the models for 30-day, 1-year, and 5-year mortality were trained on 84,239, 78,340, and 42,742 patients and evaluated on 56,059, 49,748, and 21,796 patients respectively (Supplementary Fig. 1). In the lab evaluation set, 4,907 (8.8%), 9,668 (19.4%), and 8,352 (38.3%) had died at the above-mentioned time-points, respectively.

### Model Comparisons

**Fig. 2:**
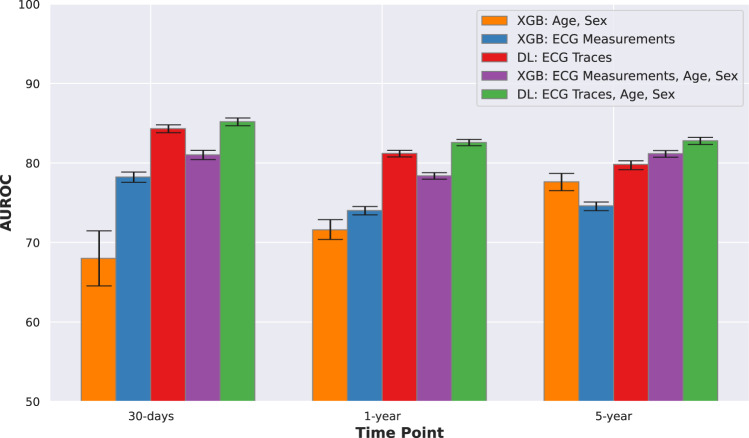
Comparison of model performances. Comparison of AUROC model performances for DL and XGB models with ECG traces and measurements. Error bars show 95% confidence intervals around the mean. DL with ECG traces, age and sex was the best model in this comparison, with AUROCs consistently higher than 0.82. AUROC Area under the receiver operating characteristic curve; DL deep learning; ECG electrocardiogram; XGB XGBoost.

The comparisons of model performances are presented in Fig. 2 and Table 3. We used age and sex features alone to establish a baseline model performance, which had an area under the receiver operating characteristic curve (AUROC) (mean and 95% confidence interval) of 0.680 (0.646–0.715) for 30-day, 0.716 (0.704–0.723) for 1-year and 0.776 (0.765–0.787) for 5-year mortality. The deep learning (DL) model with ECG traces alone had a significantly higher performance with AUROC of 0.843 (0.838–0.848), 0.812 (0.808–0.816), and 0.798 (0.792–0.803) for 30-day, 1-year, and 5-year prediction, respectively (DeLong Test, all *p* < 0.001). Using age and sex along with ECG traces showed further small but significant improvements with AUROC of 0.852 (0.847–0.857), 0.826 (0.822–0.830) and 0.828 (0.824–0.832) for the three time points (DeLong Test, all *p* < 0.001). DL with ECG traces showed significantly better performance than XGBoost (XGB) with ECG measurements for all the three time points (DeLong Test, all *p* < 0.001). DL with ECG traces, age and sex was the best model in this comparison, with AUROCs consistently higher than 0.82. XGB with ECG measurements did not perform better than just age and sex for 1-year (DeLong Test, *p* = 0.57), and was significantly worse than just age and sex for 5-year (DeLong Test, *p* < 0.001); however DL with ECG traces still provided relevant information to the prediction and significantly outperformed the baseline age and sex model at all time-points (DeLong Test, *p* < 0.001)—thereby emphasizing the prognostic utility of DL models based on ECG traces over typical models using ECG measurements. Table 3 shows the superior performance of DL models with ECG traces in terms of area under the precision-recall curve (AUPRC), F1-Score, Brier Score, and other measures.

**Table 3:** Evaluation of various model performances expressed in mean (95% confidence interval) percentage.

Time-point	Features	Model	AUROC	AUPRC	F1 Score	Specificity	Recall	Precision	Accuracy	Brier Score
30-days	Age, Sex	XGB	67.99 (64.54–71.47)	2.95 (2.16–4.06)	3.99 (3.37–4.59)	60.52 (59.86–61.11)	66.38 (60.38–72.32)	2.06 (1.73–2.37)	60.59 (59.93–61.21)	1.55 (1.41–1.68)
	ECG	DL	84.32 (83.83–84.82)	34.54 (33.24–35.81)	34.67 (33.83–35.46)	80.25 (79.96–80.56)	71.9 (70.76–73.08)	22.85 (22.17–23.46)	79.63 (79.33–79.92)	5.97 (5.83–6.12)
		XGB	78.24 (77.59–78.87)	22.07 (21.14–22.96)	30.09 (29.14–31.01)	84.54 (84.21–84.82)	51.38 (49.8–52.87)	21.27 (20.53–22.01)	82.05 (81.74–82.33)	6.44 (6.29–6.61)
	ECG, Age, Sex	DL	85.19 (84.7–85.68)	35.6 (34.31–36.84)	36.25 (35.37–37.09)	81.75 (81.45–82.05)	71.82 (70.67–73.07)	24.24 (23.56–24.93)	81.0 (80.72–81.29)	5.75 (5.62–5.89)
		XGB	81.02 (80.45–81.61)	25.72 (24.57–26.75)	32.66 (31.67–33.67)	84.71 (84.43–84.98)	56.23 (54.81–57.81)	23.02 (22.19–23.83)	82.57 (82.29–82.83)	6.28 (6.12–6.44)
1–year	Age, Sex	XGB	71.59 (70.39–72.89)	22.59 (20.93–24.19)	28.14 (26.89–29.38)	64.62 (63.97–65.32)	67.23 (64.98–69.36)	17.8 (16.89–18.69)	64.89 (64.28–65.55)	9.21 (8.91–9.46)
	ECG	DL	81.2 (80.77–81.61)	48.17 (47.11–49.11)	50.31 (49.62–51.01)	78.86 (78.54–79.18)	67.46 (66.56–68.32)	40.12 (39.45–40.82)	76.88 (76.61–77.17)	11.44 (11.3–11.6)
		XGB	74.02 (73.48–74.53)	35.92 (35.02–36.83)	41.88 (41.16–42.61)	80.88 (80.54–81.22)	50.62 (49.68–51.55)	35.72 (34.98–36.42)	75.63 (75.28–75.99)	12.86 (12.69–13.03)
	ECG, Age, Sex	DL	82.58 (82.18–82.97)	51.21 (50.33–52.22)	52.04 (51.29–52.75)	80.41 (80.1–80.71)	68.0 (66.98–68.94)	42.14 (41.43–42.83)	78.26 (77.97–78.56)	11.21 (11.05–11.37)
		XGB	78.39 (77.98–78.79)	42.03 (41.03–43.05)	46.53 (45.88–47.23)	81.96 (81.66–82.24)	56.39 (55.56–57.28)	39.61 (38.93–40.29)	77.52 (77.21–77.81)	12.19 (12.03–12.36)
5–years	Age, Sex	XGB	77.63 (76.53–78.7)	59.03 (56.73–61.62)	57.32 (55.78–58.98)	72.52 (71.35–73.6)	69.19 (67.34–70.99)	48.93 (47.19–50.89)	71.6 (70.61–72.59)	18.49 (18.13–18.86)
	ECG	DL	79.82 (79.16–80.29)	65.88 (64.89–66.74)	63.14 (62.45–63.78)	78.37 (77.89–78.84)	66.15 (65.26–66.88)	60.4 (59.68–61.17)	74.3 (73.84–74.73)	17.52 (17.29–17.83)
		XGB	74.61 (74.02–75.1)	56.21 (55.15–57.08)	54.78 (53.99–55.56)	81.04 (80.56–81.5)	52.07 (51.22–53.01)	57.78 (56.83–58.57)	71.4 (70.9–71.88)	19.05 (18.85–19.28)
	ECG, Age, Sex	DL	82.8 (82.35–83.24)	70.02 (69.17–70.91)	66.5 (65.84–67.18)	80.18 (79.69–80.63)	69.61 (68.85–70.36)	63.66 (62.86–64.48)	76.67 (76.22–77.1)	16.57 (16.34–16.81)
		XGB	81.18 (80.74–81.58)	67.26 (66.37–68.05)	63.84 (63.22–64.49)	81.68 (81.22–82.12)	64.12 (63.31–64.91)	63.56 (62.8–64.27)	75.84 (75.41–76.21)	16.5 (16.31–16.72)

*AUPRC* area under the precision-recall curve, *AUROC* area under the receiver operating curve, *DL* deep learning, *ECG* electrocardiogram, *XGB* XGBoost.

#### Risk groups

**Fig. 3:**
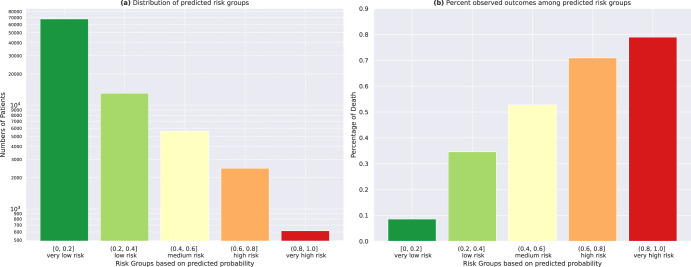
Predicted risk groups for 1-year mortality. **a** We derived five risk groups - ‘very low’, ‘low’, ‘medium’, ‘high’, ‘very high’ risk groups based on 20 percent cut-points (0 - 20%, 20% - 40%, etc.) of predicted probability of death from our main models (DL: ECG Trace, age, sex) in the holdout set. **b** Percentage of observed deaths in each predicted risk group showed good calibration with a steady increase across the risk groups (8.6%, 34.6%, 52.3%, 70.9%, and 78.9% death in the ‘very low’, ‘low’, ‘medium’, ‘high, and ‘very high’ risk groups, respectively). DL deep learning, ECG electrocardiogram.

We derived five risk groups—‘very low’, ‘low’, ‘medium’, ‘high’, ‘very high’ risk groups based on 20 percent cut-points (0–20%, 20–40%, etc.) of predicted probability of death from our main models (DL: ECG Trace, age, sex) in the holdout set (Fig. 3 for 1-year mortality, Supplementary Fig. 2 for other time points). Percentage of observed deaths in each predicted risk group showed good calibration with a steady increase across the risk groups (8.6%, 34.6%, 52.3%, 70.9%, and 78.9% death in the ‘very low’, ‘low’, ‘medium’, ‘high, and ‘very high’ risk groups, respectively).

### Model performance in diagnostic and sex subgroups

**Fig. 4:**
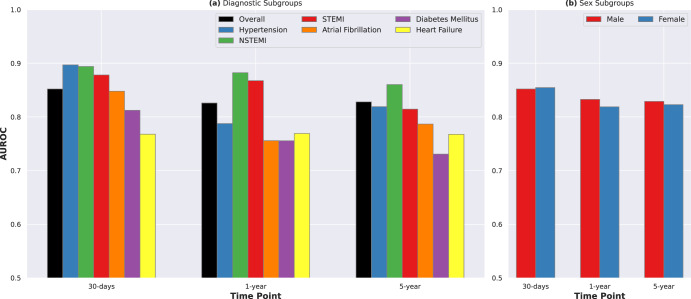
Model performances in diagnostic and sex-based subpopulations. **a** Performance of DL: ECG traces, Age, Sex models in different primary diagnosis subgroups. The models performed better in patients with STEMI and NSTEMI (AUROC of 0.867 and 0.882 for 1-year mortality, respectively) than in the overall cohort. The performance of the model in the other subgroups (heart failure, diabetes and atrial fibrillation) was lower than in the overall holdout cohort. **b** The prognostic models performed slightly better in men than in women. AUROC Area under the receiver operating characteristic curve, DL deep learning, ECG electrocardiogram, NSTEMI non-ST elevation myocardial infarction, STEMI ST elevation myocardial infarction.

Mortality rates differed significantly across the diagnostic groups of interest (Supplementary Fig. 3) with patients with heart failure having the highest mortality at each time point. Figure 4 shows the performance of our models in these different diagnosis subgroups. The models performed better in patients with ST-Elevation Myocardial Infarction (STEMI) and Non-ST-Elevation Myocardial Infarction (NSTEMI) (AUROC of 0.867 and 0.882 for 1-year mortality, respectively) than in the overall cohort. The performance of the model in the other subgroups (heart failure, diabetes, and atrial fibrillation) was lower than in the overall holdout cohort. Mortality rates were higher among men than women (Supplementary Fig. 4). In general, the prognostic models performed slightly better in men than in women (Fig. 4).

### Model performance with addition of lab features

**Fig. 5:**
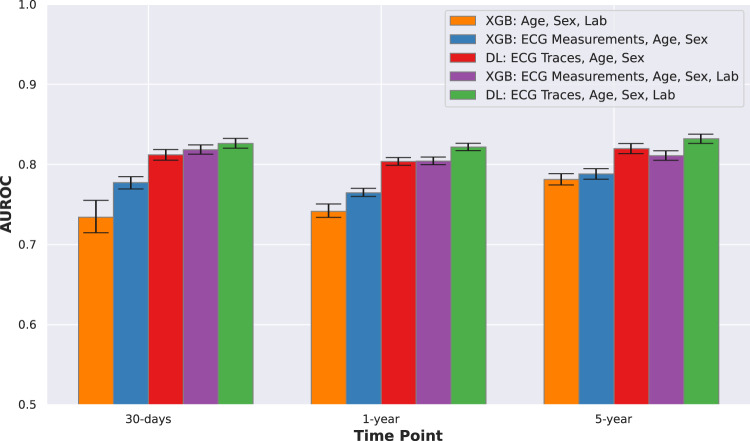
Comparison of model performances with and without lab features. Error bars show 95% confidence intervals around the mean. ECG models had higher AUROCs than the baseline model (XGB: Age, Sex, Lab) for all time points, even without lab values, but the difference was smaller for longer range predictions. Addition of lab features significantly improved the model performances throughout, across models and time points, however the gains in performance were small in magnitude (0.99% on average, DeLong Test, all *p* < 0.001). Overall, the DL model with ECG traces, age, sex and lab was the best performing model in the comparison. AUROC Area under the receiver operating characteristic curve, DL deep learning, ECG electrocardiogram, XGB XGBoost.

We explored the improvement of model performance with addition of lab features to ECG models. We considered XGB with age, sex, and lab features as the baseline model for comparison. ECG models had higher AUROCs than the baseline models for all time points, even without lab values, but the difference was smaller for longer range predictions. Addition of lab features significantly improved the model performances throughout, across models and time points, however, the gains in performance were small in magnitude (0.99% on average, DeLong Test, all *p* < 0.001). Again, the overall DL model with ECG traces, age, sex, and lab was the best performing model in this comparison (Fig. 5 and Supplementary Table 1).

### Model explanations

**Fig. 6:**
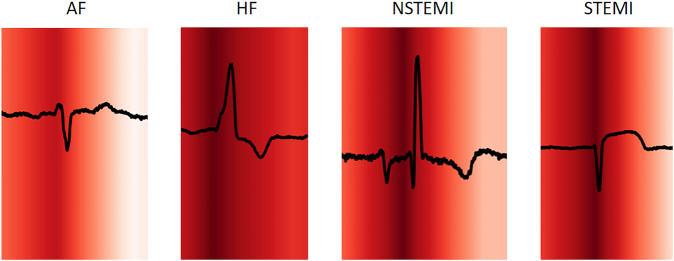
GradCAM heatmap for 1-year mortality. Representative ECG traces were chosen with primary diagnoses of AF, HF, STEMI, and NSTEMI. The darker areas in each trace on GradCAM denote the areas with the most contribution to ResNet DL: ECG model’s 1-year mortality prediction. The highlighted regions are not lead-specific, and are driven based on data from all 12 leads. AF atrial fibrillation, DL deep learning, ECG electrocardiogram, HF heart failure, STEMI ST-elevation myocardial infarction, NSTEMI Non-ST-elevation myocardial infarction.

**Fig. 7:**
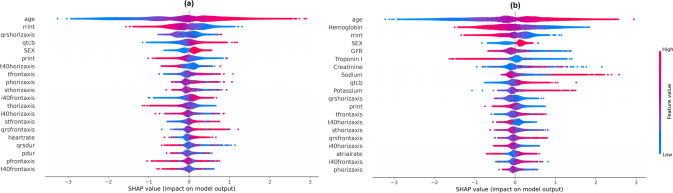
SHAP based feature importance for 1-year mortality. **a** SHAP analysis of the XGBoost models with ECG measurements, age, sex showed that higher age, lower RR interval, horizontal QRS axis (conditional effect), higher Bazett’s rate-corrected QT interval, male sex and lower PR interval contributed the most to the 1-year mortality. **b** The addition of lab features highlighted contributions of lower hemoglobin, lower glomerular filtration rate (GFR), lower troponin I, higher creatinine, very high or low sodium and high potassium. The description of ECG measurements is provided in the Supplementary Table 5.

Figure 6 depicts the results of Gradient-weighted Class Activation Mapping (GradCAM), highlighting areas of ECG with higher contribution and relevance towards the model’s mortality prediction performance. The highlighted regions are not lead-specific, and are driven based on data from all 12 leads. SHapley Additive exPlanations (SHAP) analysis of XGB model showed that higher age, lower RR interval, horizontal QRS axis (conditional effect), higher Bazett’s rate-corrected QT interval, male sex and lower PR interval contributed the most to the 1-year mortality. The addition of lab features highlighted contributions of lower hemoglobin, lower glomerular filtration rate (GFR), lower troponin I, higher creatinine, very high or low sodium and high potassium (Fig. 7 for 1-year mortality and Supplementary Fig. 5 for other time points).

### Supplementary analyses

We conducted several supplementary analyses to demonstrate the robustness of our models. First, we benchmarked the performance of our models against the custom-designed DL architecture employed by Raghunath and colleagues^14^. Training our dataset with the architecture specified in their study, resulted in an AUROC of 0.796 (0.792, 0.800) for 1-year mortality prediction, compared to 0.826 (0.822, 0.83) with our architecture. Our ResNet-based DL model showed small but statistically significant improvement in AUROC performance compared to the DL architecture employed by the Raghunath et al for all three time-points (DeLong Test, all *p* < 0.001, Supplementary Fig. 6).

Second, we evaluated our primary DL model on: (a) a holdout set which included the poor-quality ECGs that were previously excluded from the analysis cohort; and (b) a poor-quality ECG set alone (Supplementary Table 2). We found that our DL model is fairly robust to the ECG signal artifacts and acquisition issues. Addition of poor-quality ECGs (29,741 ECGs) to the original holdout set (640,527 ECGs) did not change the AUROC results (reduction of only 0.25% for 1-year mortality). Further, evaluation on poor quality ECGs alone still showed >80% on AUROC score for all three time-points. There was a 3.4%, 2.6%, and 2.5% drop in the AUROC compared to the original evaluation (which excluded poor quality ECGs) for 30-day, 1-year and 5-year time-points, respectively.

Third, to examine the performance of our models across hospitals, we conducted a leave-one-hospital out validation for each of the two tertiary hospitals (H1 and H2) among the 14 hospitals included in our study. To ensure that our training and testing sets were completely disjoint we excluded from our validation ECGs of patients who were admitted to both the training and testing hospital during the study period (Supplementary Fig. 7). We found the performance of leave-one-hospital out validation to be comparable to performance reported on the overall validation set (Supplementary Table 3). Compared to the main validation results the AUROC performance was higher by 1.27% (86.46–85.19%) for 30-day, 1.25% (83.83–82.58%) for 1-year, and 2.89% (85.69–82.8%) for 5-year models in H1 validation; but lower by 3.6% (85.19–81.59%) for 30-day, 2.41% (82.58–80.17%) for 1-year, and 1.84% (82.8–80.96%) for 5-year models in H2 validation.

Fourth, we examined the performance of our models on the entire holdout set, including multiple ECGs for holdout patients. Overall, 78,250 (80.55%), 71,636 (80.15%), and 43,629 (78.40%) patients had more than one ECG available for 30-day, 1-year, and 5-year predictions in our holdout set. The performance of our models after inclusion of multiple ECGs was comparable to their performance in the main analysis based on one random ECG per patient (Supplementary Table 4 and Supplementary Fig. 8). In addition, we found consistency in prediction across ECGs for patients with multiple ECGs (85.05%, 82.27%, and 82.25% patients had at least 50% consistently accurate predictions across their multiple ECGs at 30-days, 1-year, and 5-years, Supplementary Fig. 9).

## Discussion

Our study, based on a large, population-based cohort of patients with universal access to healthcare, demonstrates the utility of machine learning models based on ECG data to identify patients at high-risk for short- and longer-term mortality at presentation to an emergency department or hospital. We found that DL (ResNet) models based on 12-lead ECG traces perform better in predicting mortality than gradient-boosting models (XGBoost) based on routinely-reported ECG measurements. In a validation cohort of approximately 100,000 patients, ECG traces offered the most prognostic information, with the addition of patient age and sex offering small incremental improvements in model performance. Supplementary analyses demonstrated the robustness of our models’ performance in poor quality ECGs, across hospitals, and when multiple ECGs for each patient were included. Our study is the first to examine the added value of incorporating lab data, and we found that models based on the combined ECG, lab, and demographic data (patients’ age and sex) performed the best in predicting both short and long-term mortality. These findings illustrate how machine learning models can be employed to convert routinely collected data in clinical practice to knowledge that can be used to augment decision-making at the point of care as part of a learning healthcare system.

To our knowledge, only one other study has examined the prognostic utility of ECG-based machine learning models at a population-level. The study by Raghunath et al.^14^ was based on approximately 1.2 million ECGs from just over 250,000 patients, collected over a 34-year period from a single large health care system (Geisinger) in the United States. Their model for 1-year mortality based on ECG traces, age, and sex achieved an AUROC of 0.876 in a test cohort of 168,914 compared to an AUROC of 0.826 in our validation cohort consisting of 89,379 patients. There are several differences in our Canadian study and Raghunath et al.’s study from the US. Our cohort was older (average age 62.6 ± 18.6 versus 58 ± 18 years) and had significantly higher 1-year mortality rates compared to the US cohort (17.4% versus 8.4% 1-year mortality rates in the holdout sets). The higher mortality in Canada is consistent, with previously reported inter-country differences in specific patient populations and has been attributed to differences in how patients are managed in the two healthcare systems^15–17^. Raghunath et al reported a higher AUROC associated with their XGB model based on age and sex alone (0.774), while our model predicting 1-year mortality based on patient’s age and sex had an AUROC of 0.716. Our study used standard DL models (ResNet) and was based on ECGs from a single equipment manufacturer (Philips); while the US study used custom-designed DL architecture and was based on ECGs from different equipment manufacturers. Implementation of their DL architecture on our data resulted in small but significantly lower performance compared to our standard DL models, suggesting that domain shifts in the training and validation scenarios may be playing a role. Despite these significant differences in patients, health systems, equipment, and model structure, both studies found a similar degree of improvement in performance associated with the addition of ECG traces. These findings highlight the value, and potential generalizability, of ECG-based DL models for mortality prediction.

Our study extends the work by Raghunath et al.^14^ by developing models for both shorter-term (30-day) and longer-term (5-year) mortality outcomes; and examining the models’ performance in males and females separately. We found that our DL models performed consistently well at both additional time points (AUROC of 0.85 at 30-days and 0.83 at 5-years) and similarly in both sexes. However, we observed that the performance of the baseline age + sex model gets higher and closer to ECG only DL models for longer-term predictions. This suggests that while there may be clear advantages related to the application of ECG prognostication in short- to intermediate-term guiding of treatments, using ECGs alone without age and sex features might not have sufficient predictive value for 5-year mortality outcomes. Also, we found differential model performance across diagnostic subgroups, with the models performing better for patients with myocardial infarction than other disease groups.

We believe our study is the first to demonstrate the incremental prognostic value gained from including data on select lab tests. We built our models in a sequential manner, starting with just age and sex, and adding on ECG traces or measurements, and subsequently lab data. The AUROC for 1-year mortality model increased from 0.81 for the model based on age, sex, and ECG traces to 0.83 for the model based on age, sex, ECG traces, and lab. Lab data may offer more prognostic information in specific patient populations (e.g., patients with acute coronary syndromes or renal disease, etc.) and the addition of other lab measures such as AST, ALT, and HbA1c may improve our models’ performance. These examinations are being planned as part of future research studies.

DL models with convolutional neural networks are considered black boxes when it comes to identifying and interpreting patterns used by the model for prognostication. We have attempted techniques such as creating GradCAM heatmaps for that purpose, which suggest that PR intervals, QRS complexes and ST-T changes, especially the initial portion of the QRS complex contribute the most to mortality prediction. This was mostly consistent across different disease conditions, however, almost all the ECG segments had contributions to prognostication in the DL model in patients with heart failure. It should be noted that these visualization techniques are an area of active research, and it is challenging to derive clinically meaningful interpretations from ECG signals with multiple heart beats. As a complement, we used SHAP analysis for XGB models, which highlighted a few ECG parameters (such as lower RR interval, lower horizontal QRS axis, higher QT interval, and lower PR interval) which contributed the most to mortality prediction at the different follow-up periods.

Our study has some limitations. First, all ECG in our study were from the same manufacturer (Philips Intelligence System). The extent to which our findings are generalizable to ECGs from other equipment manufacturers needs to be established. Second, lab data were available only from 2012 onwards and not for all patients. One-year mortality rates were slightly higher among patients with lab data (19.4%) than among those without (17.3%). While addition of lab features resulted in small but significant improvements in model performance, it is difficult to assess whether this was related to the higher rate of adverse outcomes in this sub-group. Third, as mentioned above, the list of lab tests included in our models is not comprehensive. Fourth, our random ECG per patient cohort was slightly younger with less male patients and less comorbidities compared to the total holdout set which is attributed to more ECGs being done in older, male, and clinically-complicated patients with more comorbidities; however, we found substantial consistency in prediction across ECGs among patients with multiple ECGs in the holdout set. And lastly, ECG measurements used in XGB models were provided through Philips machines, and were not core-lab-read or human expert-curated.

In conclusion, our study demonstrates that ECG-based DL models can be used to identify patients who are at high risk for short- or longer-term mortality. These models perform equally well in males and females and can be augmented with the inclusion of data on routinely performed lab tests. Future studies are being planned to assess the utility of providing risk assessment based on ECG data in clinical practice.

## Methods

### Datasets

The province of Alberta, Canada has a single-payer (Ministry of Health: Alberta Health) and single-provider (Alberta Health Services) healthcare system. The ~4.4 million residents of the province have universal access to hospital, ambulatory, laboratory, and physician services.

For this study, ECG data were linked with the following administrative health databases using a unique patient health number: (1) the Discharge Abstract Database (DAD) containing data on hospitalizations including admission date, discharge date, most responsible diagnosis, up to 24 other diagnoses, and discharge status (transfer, discharge home, died) (2) the National Ambulatory Care Reporting System (NACRS) database of all hospital-based outpatient clinic (including emergency department) visits, which includes date of admission, most responsible diagnosis, up to 9 other diagnoses, and discharge status; (3) the Alberta Health Care Insurance Plan Registry (AHCIP), which provides demographic information (age, sex) and date of death; (4) the centralized lab data, and (5) the vital status death registry. In case of conflicting mortality status or dates (1.1% of patients), the vital status registry was given priority over the DAD, NACRS, and AHCIP registry records.

This study was approved by the University of Alberta Research Ethics Board (Pro00120852). The ethics panel determined that the research is a retrospective database review for which subject consent for access to personally identifiable health information would not be reasonable, feasible, or practical.

### ECG data

The study used standard 12-lead ECG traces and ECG measurements from the Philips IntelliSpace ECG system. ECG traces were voltage-time series, sampled at 500 Hz for the duration of 10 s for each of 12 leads (500 ⋅ 10 ⋅ 12 voltage measurements per ECG). ECG measurements are automatically generated by the ECG machine manufacturer’s built-in algorithm (Supplementary Table 5)^18^. These latter measurements include atrial rate, P duration, RR interval, Q wave onset, Fridericia rate-corrected QT interval, heart rate, PR interval, QRS duration, QT interval, Bazett’s rate-corrected QT interval, frontal P axis, frontal QRS axis in the initial 40 ms, frontal QRS axis in the terminal 40 ms, frontal QRS axis, frontal ST wave axis (equivalent to ST deviation), frontal T axis, horizontal P axis, horizontal QRS axis in the initial 40 ms, horizontal QRS axis in terminal 40 ms, horizontal QRS axis, horizontal ST wave axis, and horizontal T axis.

### Laboratory data

Centralized lab data at the population-level are available from 2012 onwards. Data on a select set of labs including creatinine, glomerular filtration rate (GFR) calculated from creatinine, haemoglobin, potassium, sodium, and troponin I were linked with the ECG data if they occurred on the same day. Labs were selected based on their association with adverse outcomes, routine use in practice, and if they were available for a significant proportion of patients.

### Analysis cohort

The study cohort consisted of patients hospitalized at 14 sites between February 2007 and April 2020 in Alberta, Canada. Our data consisted of 2,015,808 ECGs, 3,336,091 emergency department visits, 1,071,576 hospitalizations, and 260,065 patients. Concurrent healthcare encounters for a patient (emergency department visits and/or hospitalizations) that occurred within a short period of time were considered to be transfers (for example, from emergency department to hospital admission or from community hospital to tertiary hospital) and grouped into episodes. The flowchart of the decision tree used for episode definition is outlined in Supplementary Fig. 10.

An ECG record was linked to a healthcare episode if the acquisition date was within the timeframe between the admission date and discharge date of an episode (Supplementary Fig. 11). Poor quality ECGs were identified via warning flags generated by the ECG machine manufacturer’s built-in quality algorithm for the presence of muscle artifact, AC noise, baseline wander, QRS clipping, and leads-off. After excluding the ECGs that could not be linked to any episode, ECGs of patients <18 years of age, as well as ECGs with poor signal quality, our analysis cohort consisted of 1,605,268 ECGs from 748,773 episodes in 244,077 patients. See Fig. 1 for the flowchart of study design, showing sample sizes for overall study, experimental splits and different outcomes. In supplementary analyses, we evaluated the performance of our models across different hospitals and on the poor-quality ECGs that were excluded from the main study.

Our lab analysis sub-cohort included 601,307 ECGs from 330,637 episodes of 141,017 patients for whom data on all the six labs of interest were available. On average, 41.9% of ECGs could be linked to lab tests for each fiscal year from 2012 to 2019. See Supplementary Fig. 1 for the flowchart of study design for the model incorporating lab values which shows sample sizes for overall sub-study, experimental splits and different outcomes.

### Prediction tasks

**Fig. 8:**
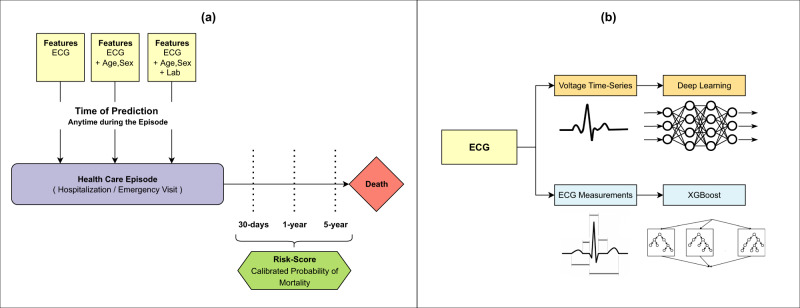
Schematic of prediction tasks, feature types and choice of learning algorithms. **a** This study focused on developing and evaluating ECG based mortality models to predict the probability of a patient dying within 30-days, 1-year and 5-years, starting from the day of ECG acquisition. ECGs used in these models could have been acquired at any time point during a healthcare episode. Models included features with **i** ECG only, **ii** ECG + age, sex, and **iii** ECG + age, sex + lab tests. The goal of the prediction models is to output a calibrated probability of mortality, which could be used in patient risk-assessment. **b** Patient’s ECG data are generally archived by healthcare facilities as one of two formats: either **i** as a clinical report of summarised ECG measurements such as QT interval, QRS duration etc. or **ii** less commonly, as raw voltage time series of ECG signal tracings. In order to facilitate wider applicability, we used learning algorithms that are appropriate for the data formats, namely ResNet based deep learning for the information-rich multi-channel voltage time series and gradient boosting-based XGBoost for the ECG measurements.

This study focused on developing and evaluating ECG based mortality models to predict the probability of a patient dying within 30-days, 1-year, and 5-years, starting from the day of ECG acquisition. ECGs used in these models could have been acquired at any time point during a healthcare episode (Fig. 8—left panel). The goal of the prediction models is to output a calibrated probability of mortality, which could be used in patient risk-assessment.

Patient’s ECG data are generally archived by healthcare facilities as one of two formats: either (a) as a clinical report of summarised ECG measurements such as QT interval, QRS duration etc or (b) less commonly, as raw voltage time series of ECG signal tracings. In order to facilitate wider applicability, we developed models to accommodate either of these ECG formats. We used learning algorithms that are appropriate for the data formats, namely ResNet based DL for the information-rich voltage time series and gradient boosting-based XGBoost (XGB) for the ECG measurements (see Fig. 8, right panel and ‘learning algorithms’ below). XGB was chosen for its state-of-art performance with structured tabular data, fast training time, missing value support, and explainability functions^19^. Likewise, we used ResNet architecture for DL based on its successful performance in previous studies with comparable datasets of ECG tracings^20^. In supplementary analysis, we benchmarked the performance of our standard DL models against custom-designed DL architecture used by a previous study which utilized deep convolutional neural networks (DNN) using five branches to accommodate varying durations of ECG acquisition across the leads^14^.

In order to examine the incremental predictive value that demographic (age, sex), and lab data add to the performance of models trained on ECGs only, we developed the models in the following sequential manner: (a) ECG only, (b) ECG + age, sex and (c) ECG + age, sex + lab, where ECG data could be either voltage-time traces or measurements.

ECGs of patients that were censored before the 30-day, 1-year, and 5-year time-points were excluded from analysis as their death status was uncertain and those that were censored after the time-points were considered as ‘alive’. For training, ECGs of patients with events, regardless of completeness of follow-up, were retained to maximize learning (e.g., for 5-year mortality, we retained ECGs of patients who entered the cohort after 2015-03-31 but died before 2020-03-31). However, for evaluation, all ECGs without complete follow up were excluded, irrespective of their death or censoring status (i.e., for 5-year mortality, we excluded all ECGs after 2015-03-31 in the evaluation set, as they would not have had the requisite five years of follow-up). The number of ECGs, episodes and patients used for modeling each time point in overall data and in experimental splits are presented in Fig. 1 and Supplementary Table 6.

### Learning algorithms

We employed a classification methodology with binary labels, i.e., dead or alive within 30 days, 365 days (1 year), or 1825 days (5 years) of ECG acquisition date respectively to estimate the probability of a new patient surviving at least 30 days, 365 days, 1825 days following the ECG acquisition. Since the input for the models that used ECG measurements was structured tabular data, we trained gradient boosted tree ensembles (XGB) models, whereas we used deep convolutional neural networks for the models with ECG voltage-time series traces^19,20^. For both XGB and DL models, we used 90% of the development data to train the model, and used the remaining 10% as a tuning set to track the performance loss and to “early stop” the training process, to reduce the chance of overfitting (different from holdout data)^21^.

The XGB model used log-likelihood as the objective function. The hyperparameters such as maximum tree depth, minimum child weight and scale positive weight were tuned based on 5-fold grid-search internal cross validation within the training sets. The models were learnt for a maximum of 200 epochs, and the learning process was stopped if performance loss in the tuning set did not reduce for 10 consecutive epochs.

For the DL model, we implemented a convolutional neural network (CNN) based on the residual neural network architecture^22^, consisting of a convolutional layer, 4 residual blocks with 2 convolutional layers per block, followed by a dense layer (total of 6,400,433 model parameters). We used batch normalization, ReLU, and dropout after each convolutional layer. Our architecture was based on a previously published large-scale study to identify abnormalities in 12-lead ECGs with some modifications to accommodate tabular data input and mortality output (Supplementary Fig. 12)^20^. Each ECG instance was loaded as a 12 × 4096 numeric matrix. If additional features such as age, sex or lab features were used, they were input as binary feature (sex; 1 feature) or continuous values (age, lab features; 1 + 6 features), then passed to a 5N-hidden-unit layer (where N is number of tabular features), then concatenated with the dense layer, and finally passed to a sigmoid function to produce the output. Binary cross-entropy was used as the loss function with the initial learning rate of 1 × 10^3^, Adam optimizer^23^, ReLU activation function, kernel size of 16, batch size of 512, and dropout rate of 0.2 with other hyperparameters set to default. Models were learnt for a maximum of 50 epochs. The learning rate was reduced to 1 × 10^−5^ if there was no improvement in tuning loss for seven consecutive epochs, and the learning process was stopped if loss in the tuning set did not reduce for nine epochs. The models were implemented using Tensorflow 2.2 and XGBoost 1.5.1 in Python 3.8. We trained all our models on the NVIDIA Driver version 418.88 with 8 Tesla V100-SXM2 GPUs and 32 GB of RAM per GPU. Each DL model took approximately 30 min to train per epoch.

### Evaluation and visualization

#### Evaluation metrics

We divided the overall ECG dataset into a random split of 60% for the model development (for training (90% subset of the 60%) and fine tuning (10% subset of the 60%) the model), and the remaining 40% as the holdout set for validation (Fig. 1 and Supplementary Table 6 for main model without lab, Supplementary Fig. 1 for secondary model with lab). We ensured that ECGs from the same patient were not shared between the development and evaluation data set. We reported the following performance metrics on the holdout set—area under the receiver operating characteristic curve (AUROC, also known as C-index) and area under the precision-recall curve (AUPRC). Also, we binarized prediction probabilities into dead/alive classes using optimal cut-points derived from training set Youden’s index^24^, and generated F1 Score, Specificity, Recall, Precision and Accuracy. Further, we evaluated calibration of our models to see whether predicted probabilities agree with observed proportions using Brier Score (baseline value is 25%; smaller score indicates better calibration)^25^.

#### Evaluation sampling

Patients with more severe illnesses are expected to undergo ECGs more frequently, and more ECGs may be acquired at terminal stages when patients are monitored more regularly. This variability in timing and frequency of ECGs across patients could lead to potential bias in model performance due to differential representation of patient phenotypes. To mitigate such bias, we evaluated our models using a single randomly-selected ECG per patient from their multiple episodes in the holdout set (Fig. 1 and Supplementary Table 6 for main model without lab, Supplementary Fig. 1 for secondary model with lab). This sampling strategy could be considered to be more representative of deploying the model in a real-world scenario on novel ECG from an unseen patient, rather than using the most recent ECG or the ECG that was taken the closest to the patient’s death (see ‘Model Comparisons’ section for details). In supplementary analyses, we examined our models’ performance on the entire holdout set, including multiple ECGs per patient, as well as examined the consistency of prediction across ECGs for patients with multiple ECGs.

#### Sex and diagnostic subgroups

We investigated our models’ performance in specific patient subgroups, based on diagnoses of interest and patient sex, in our holdout set. The diagnostic subgroups were based on the most responsible diagnosis assigned at discharge and included the following: non-ST-elevation myocardial infarction (NSTEMI), ST-elevation myocardial infarction (STEMI), heart failure, atrial fibrillation, diabetes mellitus, and hypertension. The ICD codes used for identifying these conditions are provided in Supplementary Table 7.

#### Model comparisons

For each evaluation, we used 10 iterations of random selection of a single ECG per patient to demonstrate consistency in the model performance. During the training of these models, we used all available ECGs in the training set along with corresponding mortality labels. Same training and testing splits (including the random selections) were used for the various modeling scenarios, so that performance could be compared directly. The performance scores were compared between models by bootstrapping 100 instances with sampling with replacement from each of 10 iterations of random ECG selection mentioned above, to generate a total of 1000 bootstrap replicates (Supplementary Fig. 13). The difference in the model performances was evaluated based on the overlap of 95% confidence intervals of mean AUROC scores of the compared models. We have also reported p-value of DeLong’s test^26^ to show if the AUROCs of two models were statistically significantly different.

#### Visualizations

We used Gradient-weighted Class Activation Mapping (GradCAM) to visualize the gradient activation maps that contributed to the model’s prediction of mortality in our DL models^27^. To achieve this, the last convolutional layer that contains high-level information of the deep CNN model and representative traces from the evaluation set were selected. Also, we used SHAP (SHapley Additive exPlanations) to identify the ECG measurements that were key contributors to the average mortality prediction in the XGB models^28^.

Our study has been reported according to the Transparent reporting of a multivariable prediction model for individual prognosis or diagnosis based on artificial intelligence (TRIPOD-AI) guidelines^29^.

Role of Funding Source: The study was funded by an operating grant from the Canadian Institutes of Health Research (Grant # PJT-178158). The funding agency had no role in the study design, data analysis, result interpretation, or manuscript preparation.

## Supplementary information

Supplementary Figures and Tables

## Data Availability

The data underlying this article was provided by Alberta Health Services under the terms of a research agreement. Inquiries respecting access to the data can be made directly to them. We have included an ECG dataset that is artificially generated using variational autoencoders for the purpose of code demonstration only. They are not expected to accurately represent real ECG signals. The demo dataset is openly available, and can be downloaded at 10.6084/m9.figshare.21612786.v1.

## Code availability

The code base for training the deep learning models used in this study is available at: 10.6084/m9.figshare.21612786.v1.
