# Supplementary material for: Towards artificial intelligence-based learning health system for population-level mortality prediction using electrocardiograms
Source: NPJ Digit Med. 2023 Feb 6;6:21. doi: 10.1038/s41746-023-00765-3 (PMC9902450; doi:10.1038/s41746-023-00765-3)
Supplement: Supplementary file 1 — Supplementary Figures and Tables [file 41746_2023_765_MOESM1_ESM.pdf]

Supplementary Figures

Supplementary Figure 1: Flowchart of the study design for subset of ECGs with lab values available, showing the sample sizes for different splits and outcomes.

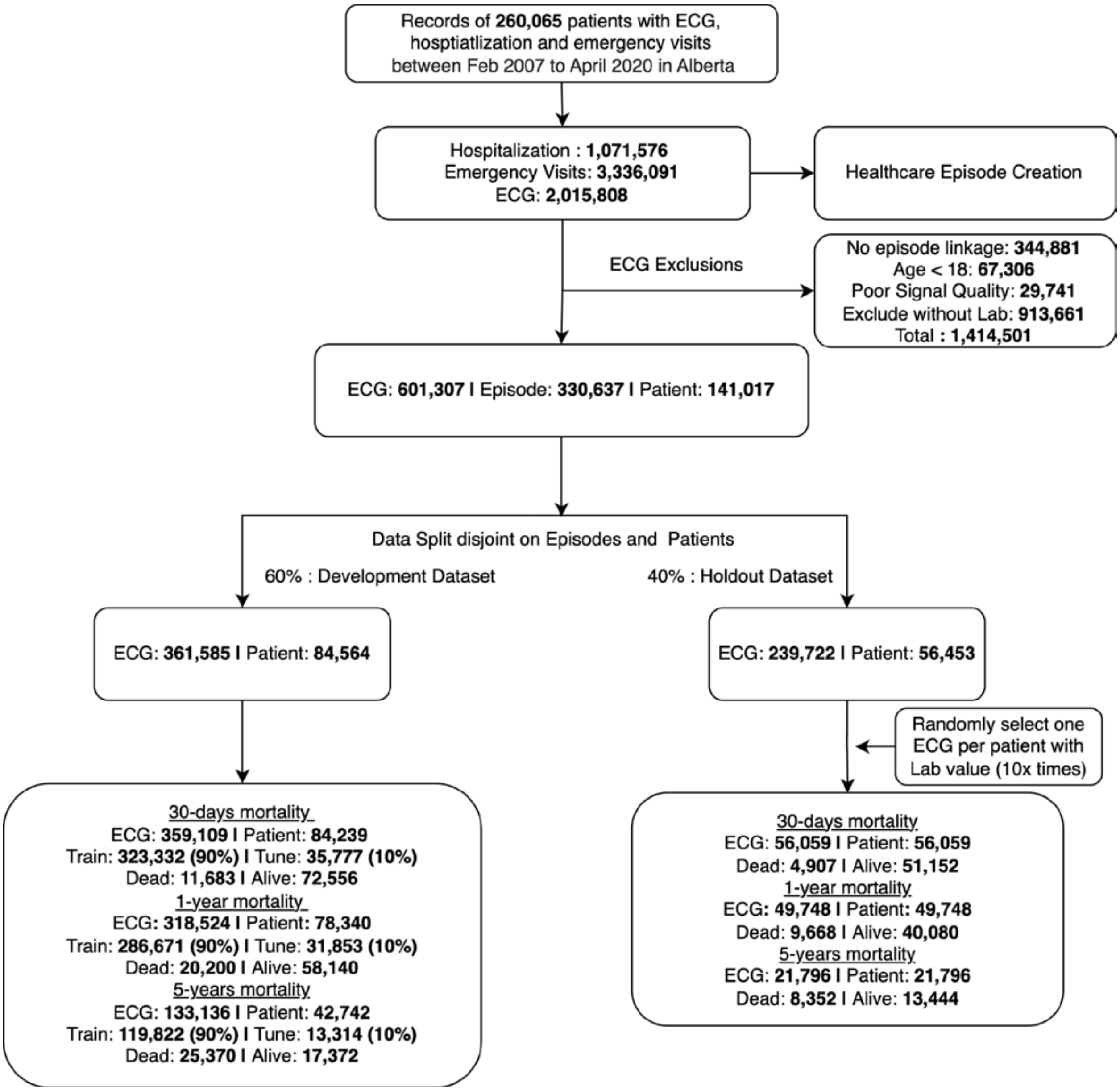

Supplementary Figure 2: Predicted risk groups in the evaluation set with DL: ECG traces, Age, Sex for the three time-points*

*Note the range of y-axis is different for the distribution of predicted risk groups in the three time-points

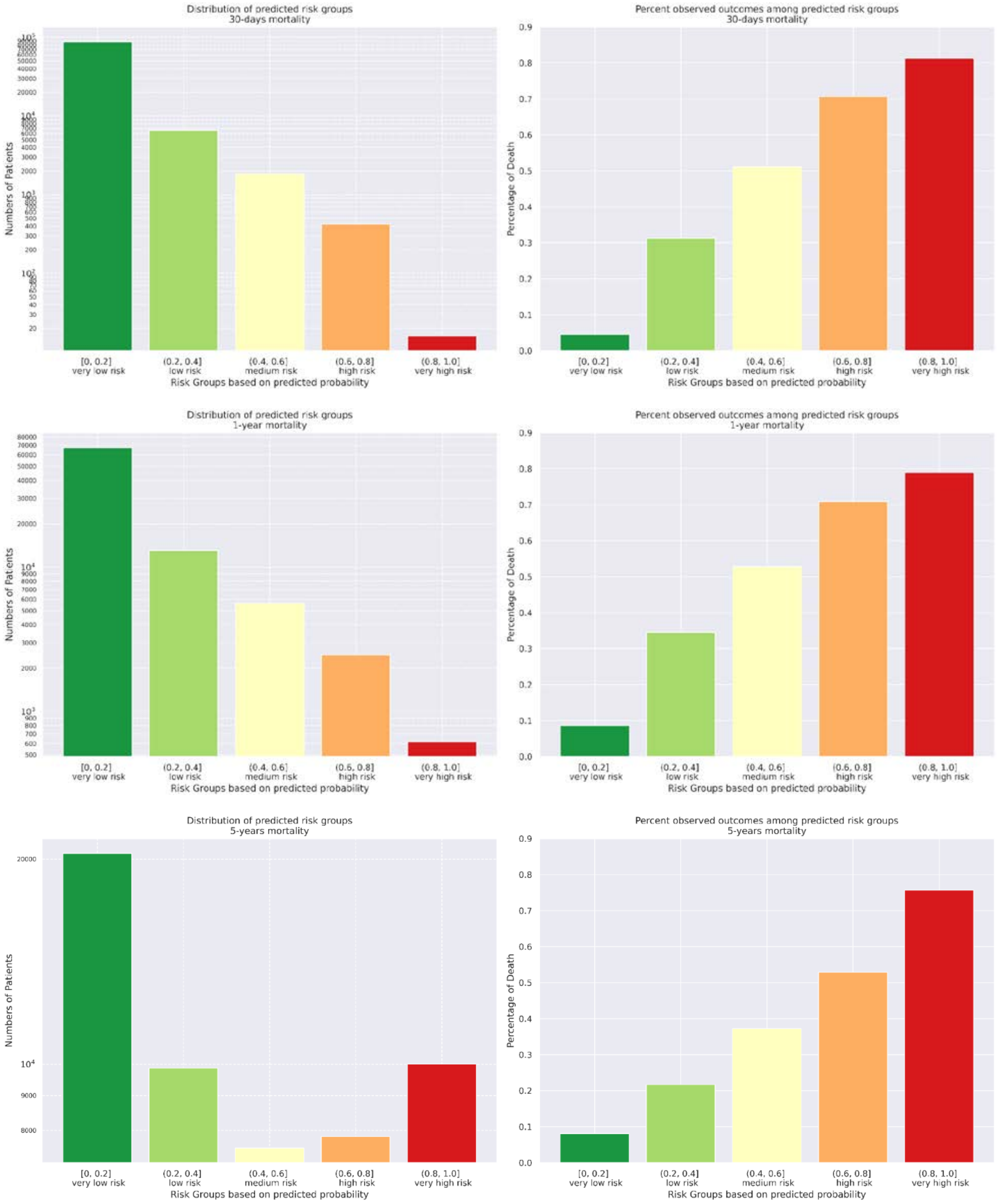

Supplementary Figure 3: Kaplan Meier curves for diagnostic subgroups in the study dataset. NSTEMI: Non ST-elevation myocardial infarction; STEMI: ST-elevation myocardial infarction;

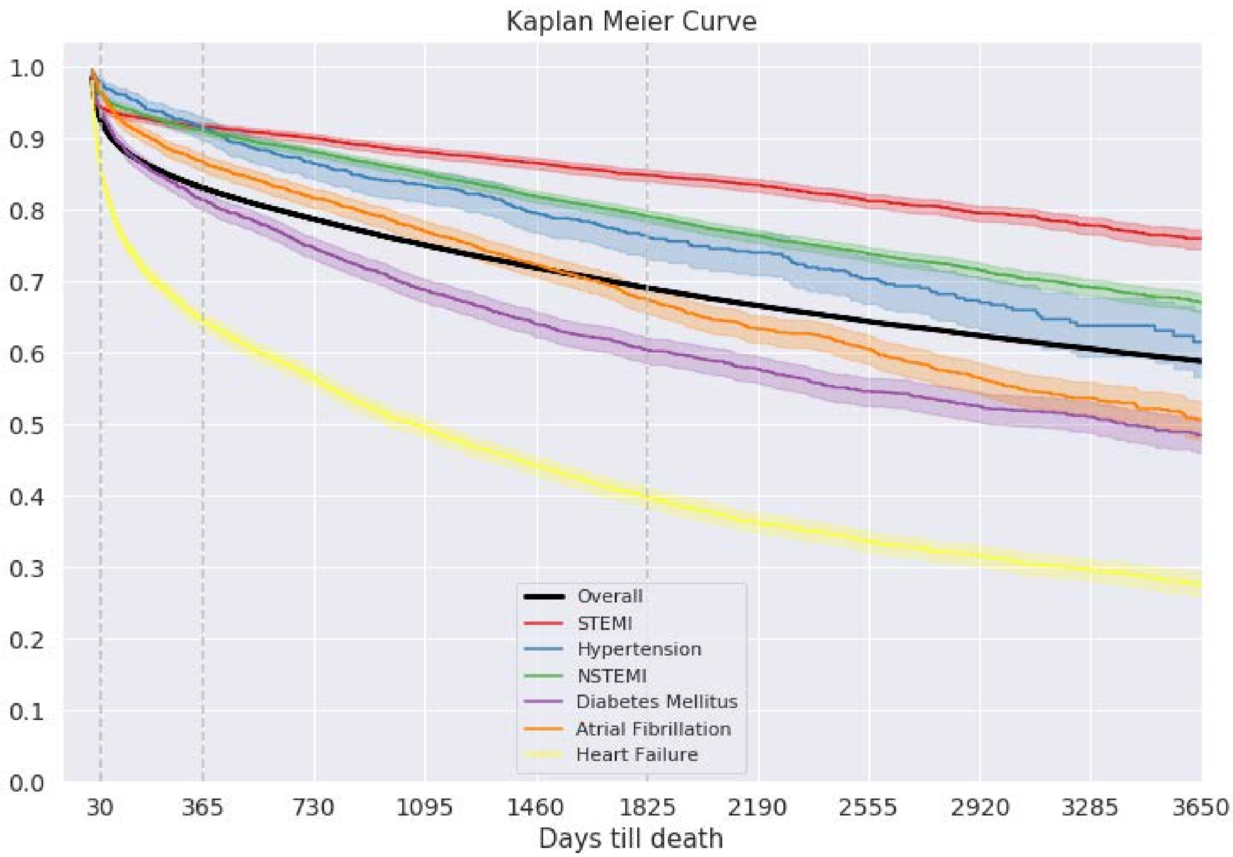

Supplementary Figure 4: Kaplan Meier curves for males and females in the study dataset

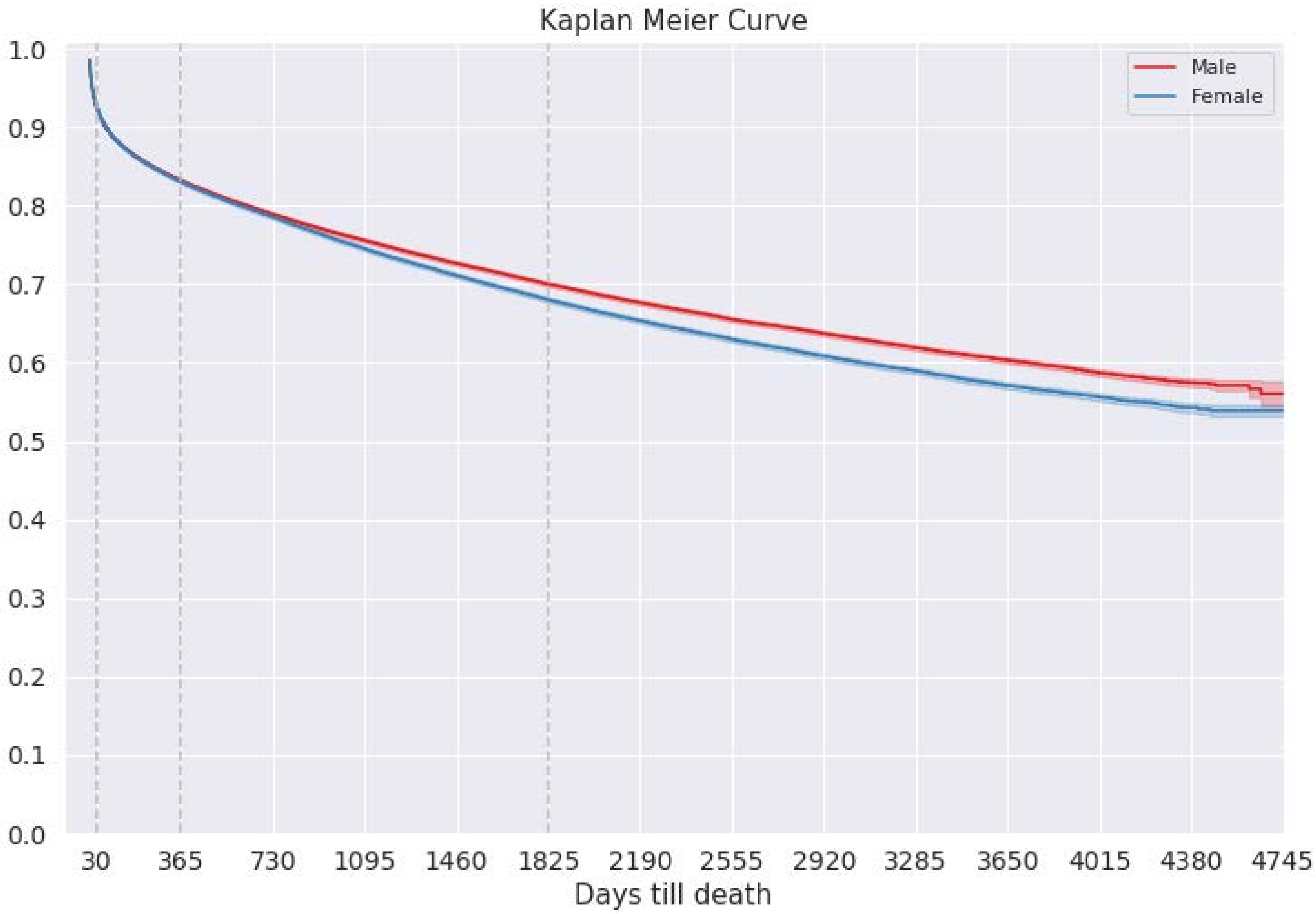

Supplementary Figure 5: SHAP based feature importance for various XGBoost models with ECG measurements, age, sex and lab features at different time-points.

- Top three predictors of 30-day mortality were were shorter RR interval, higher ST horizontal axis, and longer Q Bazett's rate-corrected QT interval in the model that included ECG measurements only; older age, shorter RR interval, and longer Bazett's rate-corrected QT interval in the model based on ECG+age and sex; and older age, lower hemoglobin level, and lower RR interval in the model based on ECG, age+sex, and labs.

- The top predictors of 1-year mortality were similar to those for 30-day mortality and included lower RR interval, higher p wave horizontal axis, and longer Bazett's rate-corrected QT interval in the ECG only model; older age, lower RR interval, and horizontal QRS axis (conditional effect) in the ECG+ age and sex model; and older age, lower hemoglobin levels and lower RR interval in the ECG, age+sex, and labs model.

- The top three predictors of 5-year mortality based on ECG measurements alone included lower Q onset, lower p wave duration (conditional effect) and higher p wave horizontal axis. "Q onset" represents the time from the start of the representative beat to the q onset in milliseconds. A lower Q onset likely represents tachyarrhythmias, which may be associated with poorer outcomes. When age and sex were included in the model, older age, lower Q onset, and lower p duration (conditional effect) were shown to be the top three predictors of 5-year mortality. Lastly, the addition of lab features found older age, lower hemoglobin, and higher creatinine as the top three predictors of 5-year mortality

	ECG Measurements	ECG Measurements, Age, Sex	ECG Measurements, Age, Sex, Lab
30-days	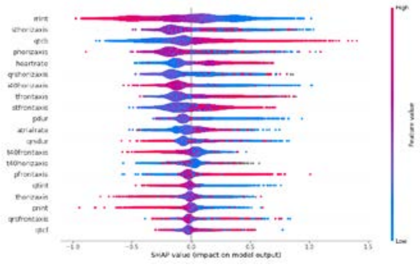	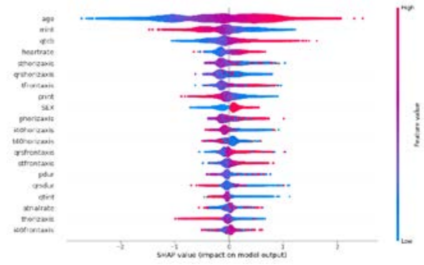	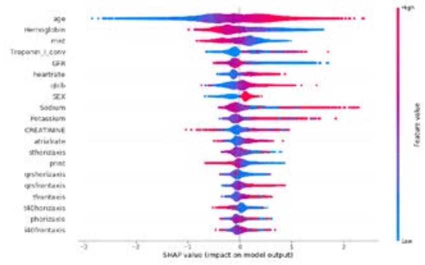
1-year	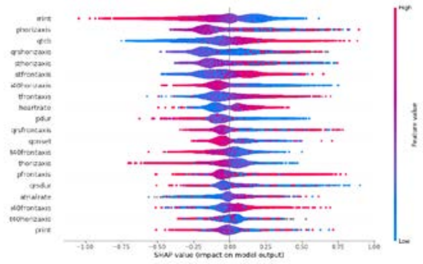	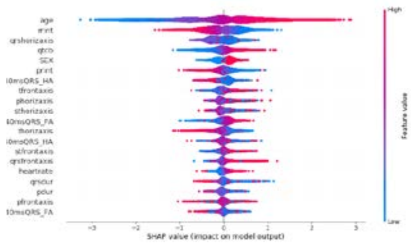	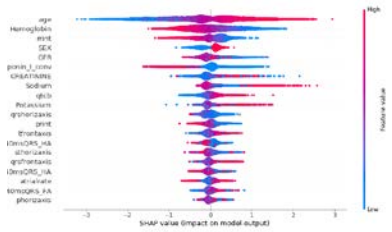
5-years	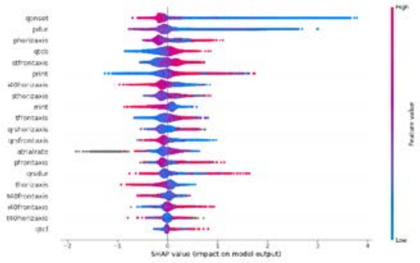	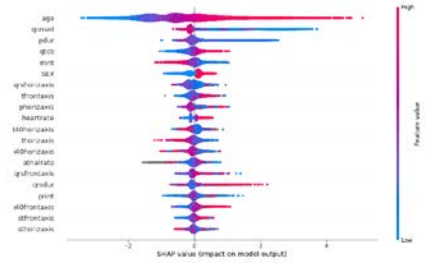	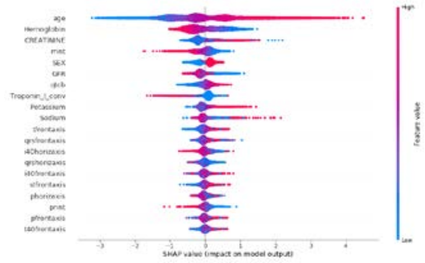

Supplementary Figure 6: Comparison of model performances between current study's XGB, ResNet DL and Raghunath et al DNN models. Error bars show 95% confidence Interval around the mean.

We benchmarked the performance of our models against those of Raghunath and colleagues. Their study used custom-designed DL architecture that utilized deep convolutional neural networks (DNN) using five branches to accommodate varying durations of ECG acquisition across the leads. We trained our dataset with the DNN model using the architecture specified in their published study.

Our ResNet based DL model showed small but statistically significant improvement in AUROC performance compared to the DL architecture employed by Raghunath et al for all three time-points. Their architecture also took a longer time to train than our ResNet DL model (Raghunath et al.: 40 to 50 epochs with ~50 min per epoch; ResNet DL: 20 to 30 epochs with ~30 min per epoch).

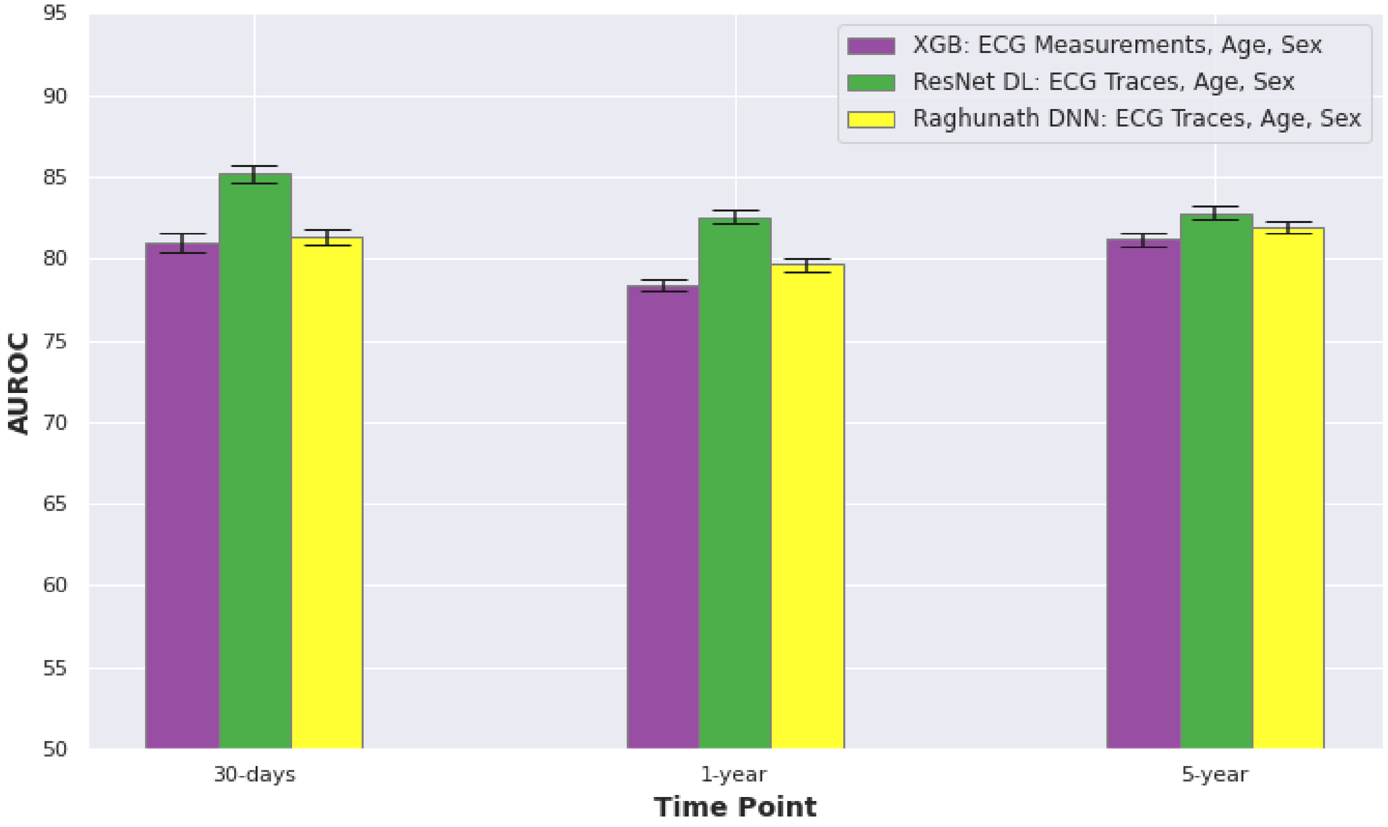

Supplementary Figure 7: Flowchart of the study design showing the sample sizes for different splits and outcomes for leave-one-hospital out validation for the Hospitals H1 (top panel) and H2 (bottom panel).

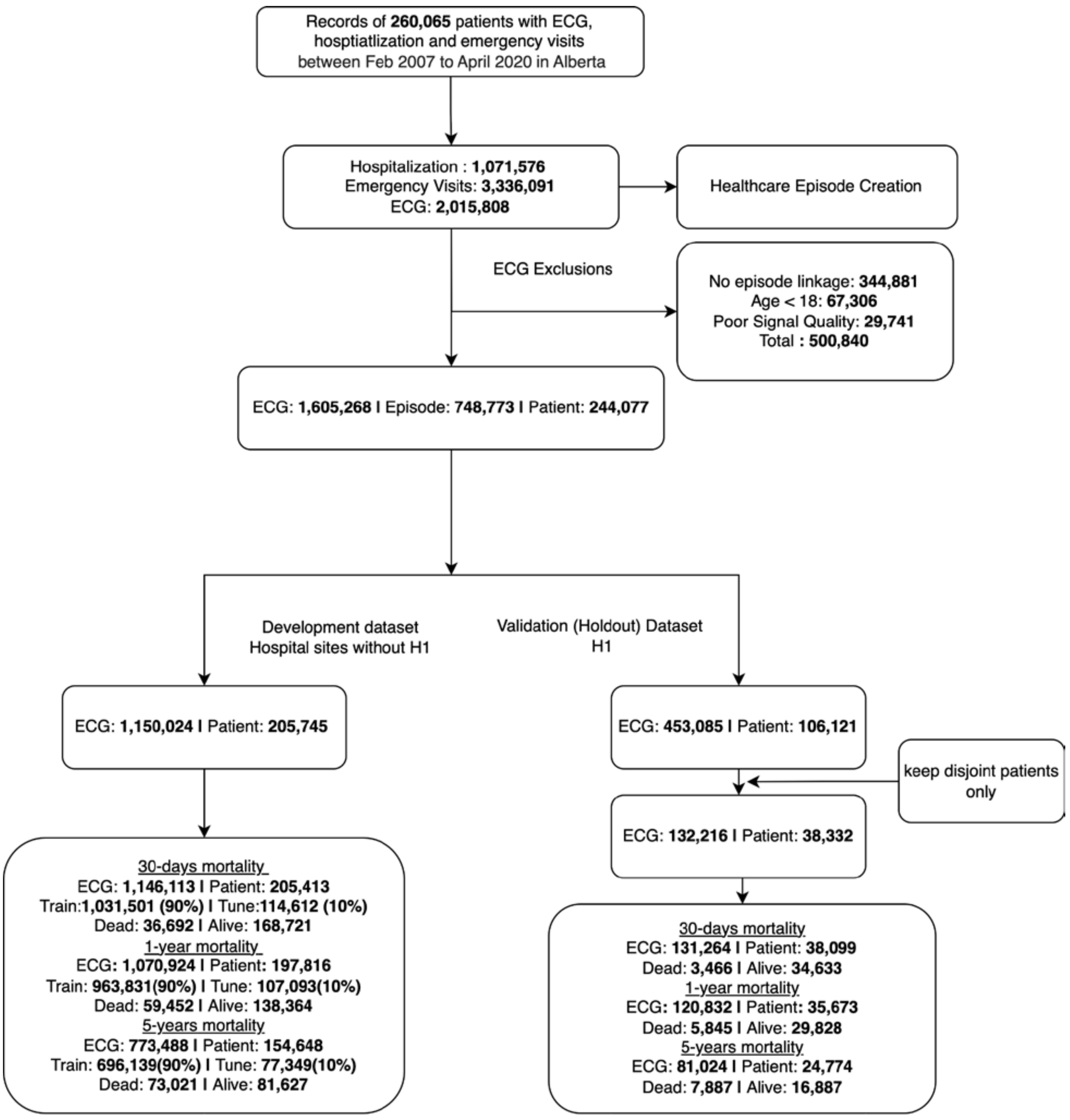

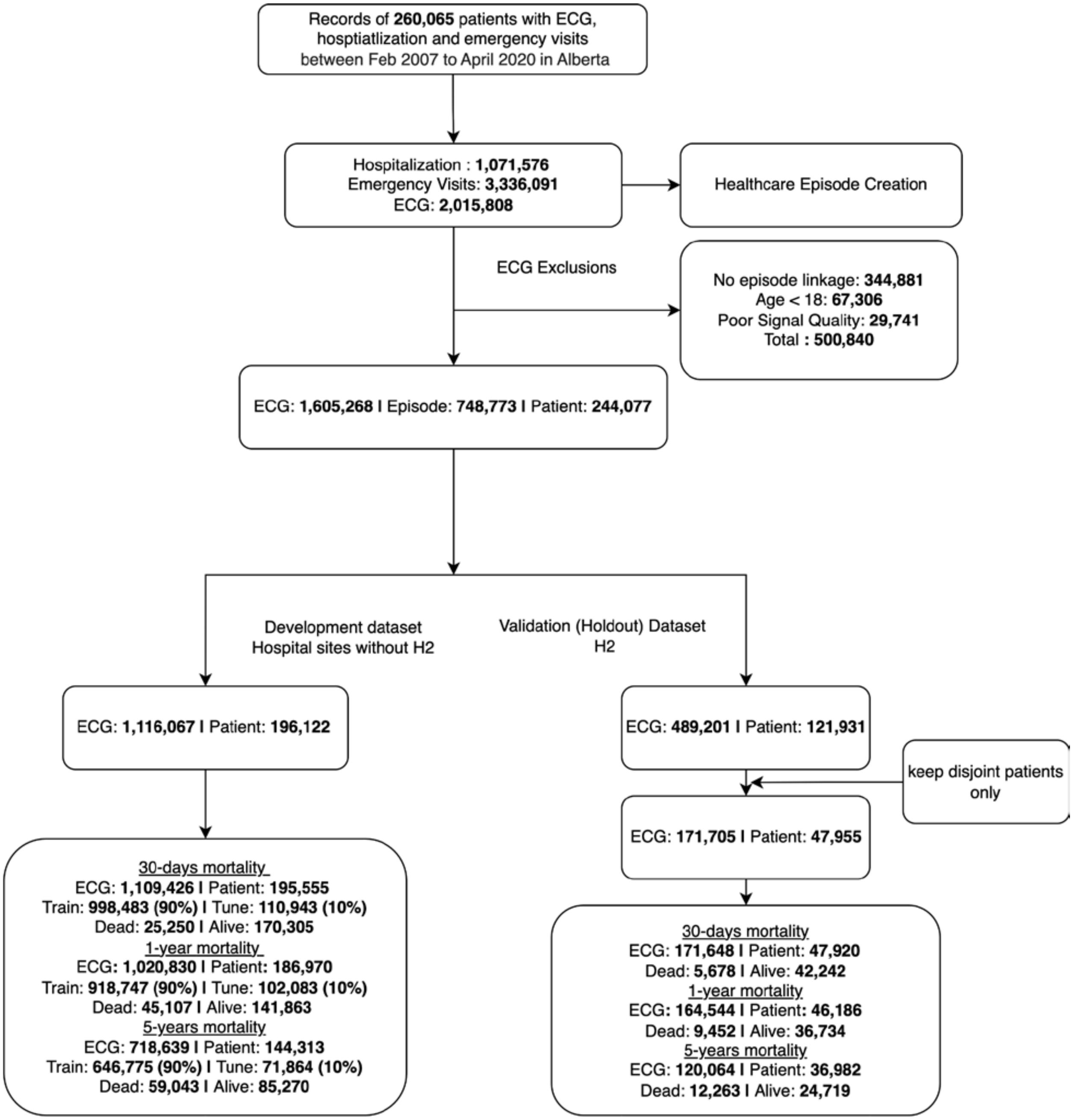
Records of 260,065 patients with ECG, hosptiatlization and emergency visits between Feb 2007 to April 2020 in Alberta
Hospitalization : 1,071,576
Emergency Visits: 3,336,091
ECG: 2,015,808
Healthcare Episode Creation
ECG Exclusions
No episode linkage: 344,881
Age < 18: 67,306
Poor Signal Quality: 29,741
Total : 500,840
ECG: 1,605,268 I Episode: 748,773 I Patient: 244,077
Development dataset
Hospital sites without H2
Validation (Holdout) Dataset
H2
ECG: 1,116,067 I Patient: 196,122
ECG: 489,201 I Patient: 121,931
keep disjoint patients only
ECG: 171,705 I Patient: 47,955
30-days mortality
ECG: 1,109,426 I Patient: 195,555
Train: 998,483 (90%) I Tune: 110,943 (10%)
Dead: 25,250 I Alive: 170,305
1-year mortality
ECG: 1,020,830 I Patient: 186,970
Train: 918,747 (90%) I Tune: 102,083 (10%)
Dead: 45,107 I Alive: 141,863
5-years mortality
ECG: 718,639 I Patient: 144,313
Train: 646,775 (90%) I Tune: 71,864 (10%)
Dead: 59,043 I Alive: 85,270
30-days mortality
ECG: 171,648 I Patient: 47,920
Dead: 5,678 I Alive: 42,242
1-year mortality
ECG: 164,544 I Patient: 46,186
Dead: 9,452 I Alive: 36,734
5-years mortality
ECG: 120,064 I Patient: 36,982
Dead: 12,263 I Alive: 24,719

Supplementary Figure 8: Comparison of AUROC model performances for DL and XGB models with ECG traces and measurements on the entire holdout set (including multiple ECGs for holdout patients)

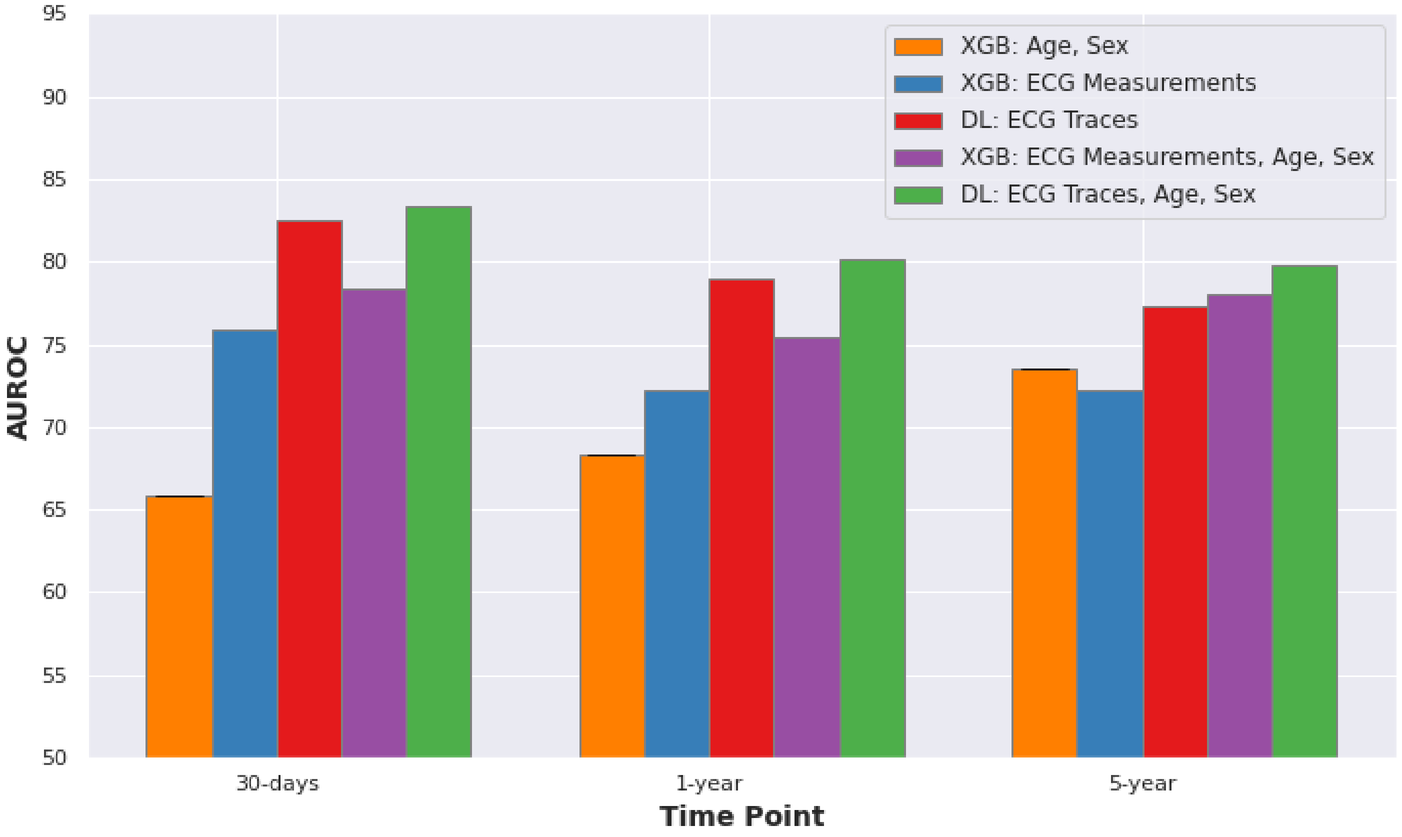

Supplementary Figure 9: Histograms for the number of patients with a certain percentage of correct predictions for their multiple ECGs for DL: ECG, age, sex models.

Consistency in the ‘correctness’ of repeat prediction for patients with multiple ECGs would indicate the robustness of our models’ performance. However, it should be noted that due to the longitudinal nature of our data, the ECGs were acquired at different time points during the course of a patient’s illness; therefore, we don’t expect the predictive probabilities of mortality to remain similar throughout.

We identified 78,250 out of 97,144 (80.55%), 71,636 out of 89,379 (80.15%), and 43,629 out of 55,650 (78.40%) patients who had more than one ECGs available for 30-day, 1-year and 5-year predictions in our holdout set. Given below are the histograms of the number of patients with correct predictions across their multiple ECGs. We found that 66,555 (85.05%), 58,935 (82.27%), and 35,883 (82.25%) patients had at least 50% consistently accurate predictions for their multiple ECGs at 30-days, 1-year, and 5-years.

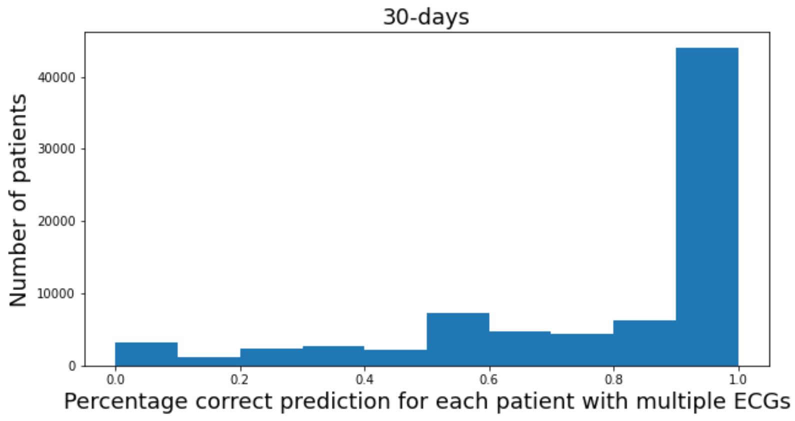

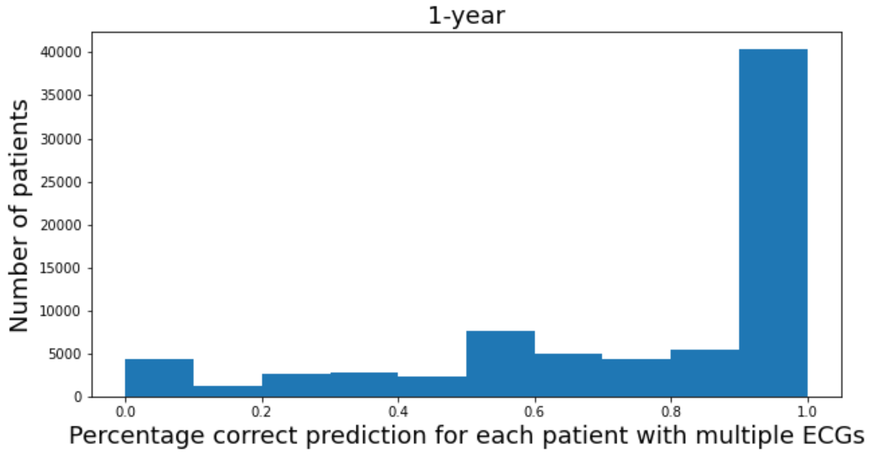

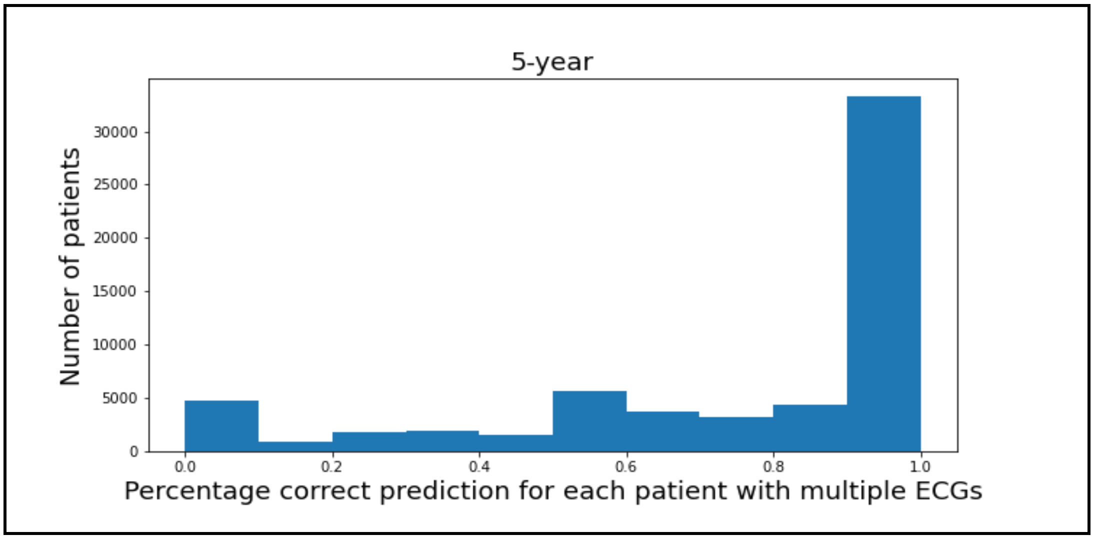
5-year
Number of patients
0
5000
10000
15000
20000
25000
30000
0.0
0.2
0.4
0.6
0.8
1.0
Percentage correct prediction for each patient with multiple ECGs

Supplementary Figure 10: Decision tree for generating healthcare episodes. Each patient can have a series of encounters over the time period which include inpatient and outpatient (including emergency department) visits. There are following scenarios where these encounters belong to the same healthcare episode: 1. Patient first visits the emergency department and then transferred to a hospital facility as an inpatient or to another emergency department within 48 hours, rather than being discharged home. 2. Patient is admitted as an inpatient in a hospital and then transferred to a different hospital facility as an inpatient or to the emergency department within 48 hours, rather than being discharged home. With our episode generation decision tree algorithm, we combine continuous healthcare visits and diagnosis codes from these encounters into a single healthcare episode. ED: Emergency department; INP: Inpatient.

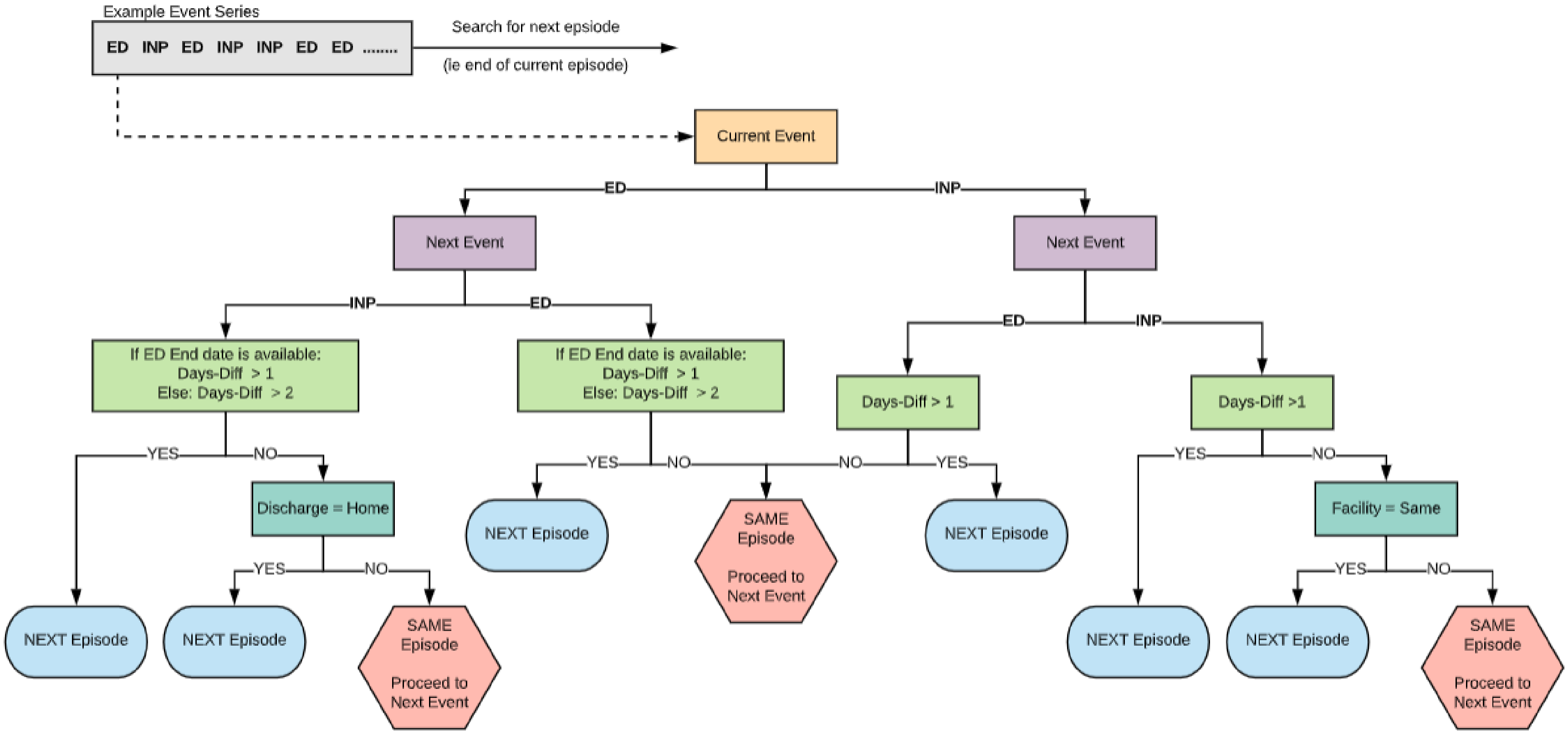

**Days-Diff: Start date of Next Event minus End date of Current Event*

Supplementary Figure 11: Date based matching of ECGs to healthcare encounters based on timeline of events for a representative patient. AMB_ED: Ambulatory / Emergency Department visit; DAD: Discharge Abstract Database for hospitalizations; EPI: Episode created based on AMB_ED and DAD; ECG: Electrocardiogram.

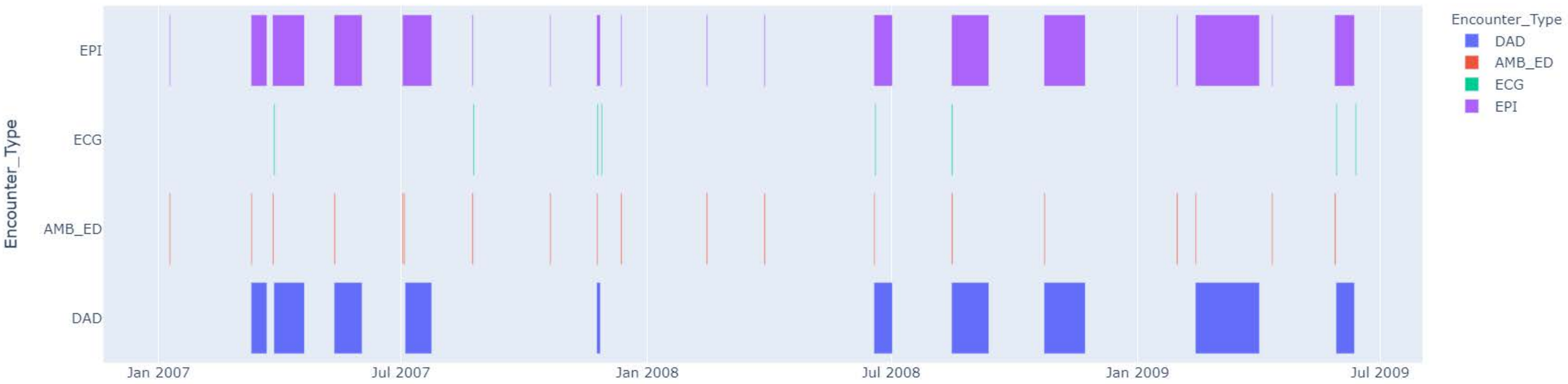

Supplementary Figure 12: Schematic of deep learning model architecture used in the study.

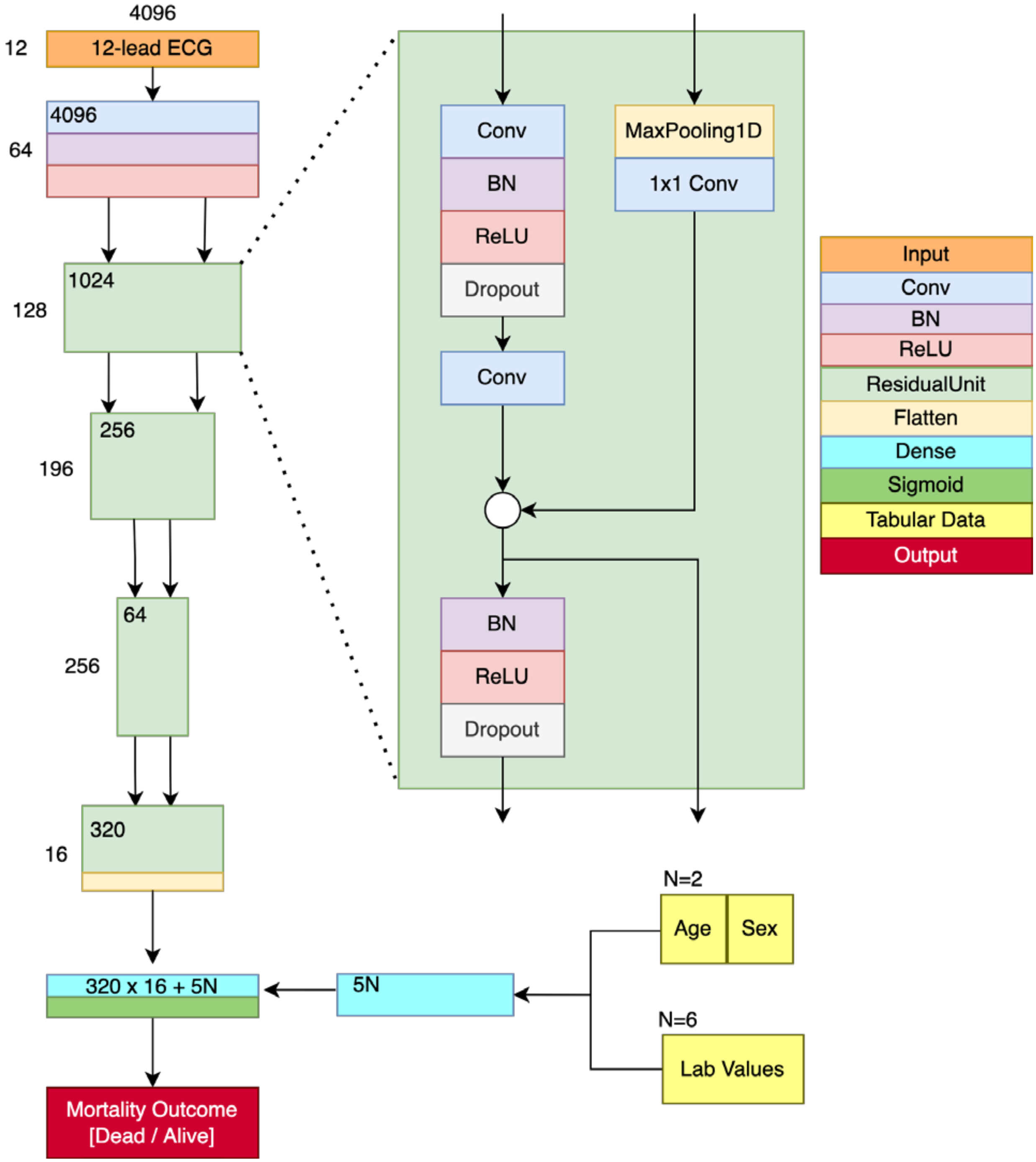

Supplementary Figure 13: Algorithm used for generating 95% confidence intervals of performance measures using bootstrap method, while avoiding the overweighting of sicker patients with disproportionately higher number of ECGs. AUROC: Area under the receiver operating curve; ECG: electrocardiogram; MLed: machine learned.

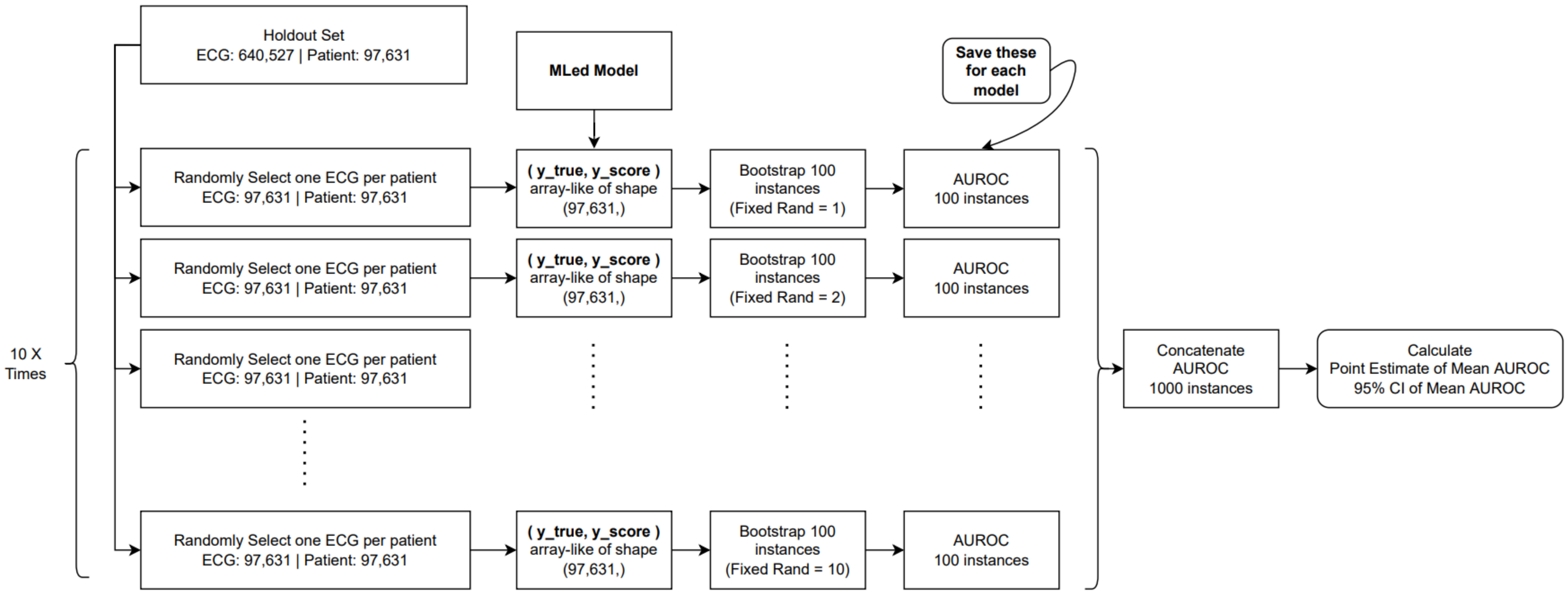

Supplementary Tables

Supplementary Table 1: Evaluation of various model performances expressed in mean (95% confidence interval) percentage using the lab subset. AUPRC: area under the precision-recall curve; AUROC: area under the receiver operating curve; DL: deep learning; ECG: electrocardiogram; XGB: XGBoost.

Time-point	Features	Model	AUROC	AUPRC	F1 Score	Specificity	Recall	Precision	Accuracy	Brier Score
**30-days**	Age, Sex, Lab	XGB	74.08 (71.32-76.82)	3.68 (2.58-5.29)	6.47 (4.93-7.91)	87.84 (87.39-88.31)	35.88 (28.65-43.6)	3.56 (2.69-4.35)	87.2 (86.74-87.68)	1.8 (1.67-1.93)
	ECG, Age, Sex	DL	81.69 (80.82-82.61)	32.86 (30.82-34.9)	33.94 (32.64-35.24)	75.04 (74.44-75.63)	72.7 (70.81-74.63)	22.14 (21.13-23.2)	74.83 (74.26-75.39)	7.14 (6.83-7.43)
		XGB	78.03 (77.03-79.01)	24.78 (23.03-26.69)	32.26 (30.72-33.79)	83.73 (83.14-84.27)	51.29 (49.15-53.44)	23.54 (22.15-24.9)	80.85 (80.27-81.36)	7.46 (7.17-7.74)
	ECG, Age, Sex, Lab	DL	82.94 (82.03-83.79)	35.11 (33.01-37.18)	37.22 (35.6-38.74)	80.69 (80.13-81.23)	68.09 (65.89-70.12)	25.61 (24.33-26.88)	79.57 (79.05-80.07)	6.91 (6.63-7.18)
		XGB	82.29 (81.38-83.16)	33.27 (31.28-35.28)	38.37 (36.42-40.32)	92.18 (91.79-92.64)	42.76 (40.55-45.12)	34.8 (32.82-36.97)	87.78 (87.32-88.3)	7.12 (6.84-7.42)
**1-year**	Age, Sex, Lab	XGB	74.9 (73.68-76.0)	23.76 (22.03-25.43)	31.73 (30.11-33.42)	82.05 (81.44-82.61)	48.58 (46.03-51.04)	23.57 (22.21-25.02)	78.63 (78.04-79.24)	9.43 (9.14-9.72)
	ECG, Age, Sex	DL	80.75 (79.95-81.49)	49.58 (47.57-51.46)	50.52 (49.19-51.83)	74.71 (74.05-75.32)	71.23 (69.56-72.75)	39.14 (37.89-40.41)	74.06 (73.48-74.61)	12.05 (11.78-12.32)
		XGB	77.02 (76.22-77.76)	41.35 (39.84-42.95)	45.8 (44.51-47.1)	81.56 (81.01-82.12)	53.7 (51.96-55.35)	39.94 (38.6-41.28)	76.38 (75.85-76.92)	13.08 (12.77-13.38)

	ECG, Age, Sex, Lab	DL	82.61 (81.79-83.36)	52.64 (50.48-54.4)	52.91 (51.62-54.23)	77.61 (77.04-78.17)	71.24 (69.66-72.83)	42.08 (40.82-43.39)	76.43 (75.88-77.01)	11.65 (11.34-11.96)
		XGB	80.88 (80.03-81.65)	48.8 (46.74-50.58)	49.92 (48.36-51.35)	85.98 (85.4-86.55)	53.68 (51.75-55.54)	46.66 (45.01-48.19)	79.98 (79.34-80.53)	12.33 (12.03-12.66)
**5-years**	Age, Sex, Lab	XGB	78.46 (77.35-79.68)	56.18 (53.85-58.6)	55.46 (53.61-57.24)	82.96 (81.92-83.96)	55.56 (53.47-57.67)	55.38 (53.21-57.61)	75.41 (74.44-76.32)	17.81 (17.3-18.33)
	ECG, Age, Sex	DL	82.13 (81.18-83.03)	70.06 (68.19-71.79)	67.45 (66.03-68.72)	75.29 (74.11-76.41)	74.01 (72.51-75.7)	61.95 (60.35-63.46)	74.84 (73.86-75.8)	18.27 (17.82-18.74)
		XGB	79.14 (77.97-80.21)	65.76 (63.51-67.75)	63.03 (61.46-64.53)	77.94 (76.79-79.07)	64.69 (62.83-66.49)	61.45 (59.63-63.34)	73.28 (72.28-74.37)	18.96 (18.4-19.56)
	ECG, Age, Sex, Lab	DL	83.31 (82.39-84.23)	72.8 (71.2-74.45)	68.39 (67.1-69.79)	78.63 (77.55-79.69)	72.4 (70.76-74.16)	64.81 (63.25-66.39)	76.43 (75.5-77.43)	17.6 (17.09-18.09)
		XGB	81.33 (80.28-82.28)	69.17 (67.18-71.06)	66.35 (64.72-67.82)	75.97 (74.85-77.13)	71.61 (69.67-73.66)	61.83 (60.16-63.35)	74.43 (73.34-75.44)	18.09 (17.52-18.69)

Supplementary Table 2: Comparative evaluation of DL: ECG, Age, Sex model performances with and without poor quality ECGs, expressed in mean percentage. AUPRC: area under the precision-recall curve; AUROC: area under the receiver operating curve; DL: deep learning; ECG: electrocardiogram; XGB: XGBoost.

Poor quality ECG may be a potential roadblock in the real-world implementation of our algorithms. In our study, 29,741 out of the total 2,015,808 ECGs were excluded based on poor quality status. This is a mere 1.47 % - hence, we expect that rejection of incoming ECGs based on quality in real world deployment to be minimal (at least in Alberta, Canada). Since our models have been trained on a large dataset of mixed quality ECG signals (i.e. consisting of minor artifacts that were not flagged by the ECG system software), we expect that performance of our DL models wouldn't degrade drastically on ECGs with poor signal quality or drop offs.

We evaluated our primary DL model on a) holdout set which now includes the poor quality ECGs that were previously excluded from the analysis cohort, and b) poor quality ECG set alone. As shown in this table, our DL model is fairly robust to the ECG signal artifacts and acquisition issues. Addition of poor quality ECGs (29,741 ECGs) to the original holdout set (640,527 ECGs) did not change the AUROC results. Further, evaluation on poor quality ECGs alone still showed >80% on AUROC score for all three time-points. There was about 3.4%, 2.6% and 2.5% drop in the AUROC compared to original evaluation (excluding any poor quality ECGs) for 30-day, 1-year and 5-year time-points respectively.

**Time- point**	**Evaluation Set**	**AUROC**	**AUPRC**	**F1 Score**	**Specificity**	**Recall**	**Precision**	**Accuracy**	**Brier Score**	**Positive N**	**Negative N**
**30-days**	Holdout Set (Random ECG per patient)	85.19	35.6	36.25	81.75	71.82	24.24	81.0	5.75	21647	75518

	Holdout Set + Poor Quality ECGs (Random ECG per patient)	84.57	34.89	35.22	79.50	73.39	23.17	79.03	5.97	8490	100822
	Poor Quality ECGs only	81.76	36.51	32.20	60.65	85.96	19.81	63.22	7.90	2543	22481
**1-year**	Holdout Set (Random ECG per patient)	82.58	51.21	52.04	80.41	68.0	42.14	78.26	11.21	23675	69693
	Holdout Set + Poor Quality ECGs (Random ECG per patient)	82.33	52.44	52.96	77.94	70.76	42.31	76.60	11.83	18877	82563
	Poor Quality ECGs only	79.96	54.03	53.25	61.70	82.36%	39.34	66.49	14.15	5221	17307
**5-years**	Holdout Set (Random ECG per patient)	82.8	70.02	66.5	80.18	69.61	63.66	76.67	16.57	22219	58385

	Holdout Set + Poor Quality ECGs (Random ECG per patient)	83.22	77.18	71.80	79.70	71.35	72.26	76.15	16.81	30724	41468
	Poor Quality ECGs only	80.35	75.99	74.08	65.46	80.91	68.31	72.87	19.53	8711	9465

Supplementary Table 3: Independent hospital evaluation of DL: ECG, Age, Sex model performances, expressed in mean percentage. AUPRC: area under the precision-recall curve; AUROC: area under the receiver operating curve; DL: deep learning; ECG: electrocardiogram; XGB: XGBoost; H1: hospital number 1; H2: hospital number 2

The study included ECGs from 14 hospitals. Among these, two were tertiary care hospitals that contributed the largest number of ECGs (453085 ECGs and 487042 ECGs, respectively). We carried out a leave-one-hospital out validation for each tertiary hospital (H1 and H2). To ensure that our training and testing sets were completely disjoint, we excluded ECGs of patients from our validation who were admitted to both the training and testing hospitals during the study period.

We found the performance of leave-one-hospital out validation to be comparable to performance reported on the overall validation set. Compared to main validation results, the AUROC performance was slightly higher by 1.27% (86.46% - 85.19%) for 30-day, 1.25% (83.83% - 82.58%), for 1-year, and 2.89% (85.69% - 82.8%) for 5-year models in H1 validation; but lower by 3.6% (85.19% - 81.59%) for 30-day, 2.41% (82.58% - 80.17%) for 1-year, and 1.84% (82.8% - 80.96%) for 5-year models in H2 validation.

Time-point	Validation Hospital	AUROC	AUPRC	F1 Score	Specificity	Recall	Precision	Accuracy	Brier Score	Positive N	Negative N
30-days	H1	86.46	39.90	40.85	81.39	75.26	28.03	80.85	6.50	11534	119730
	H2	81.59	33.22	36.44	72.30	75.92	23.97	72.67	8.08	17710	153938
1-year	H1	83.83	54.70	55.63	81.66	69.42	46.41	79.38	11.37	22502	98330
	H2	80.17	52.59	54.58	75.24	69.59	44.90	73.97	14.05	36982	127562
5-years	H1	85.69	79.53	73.53	81.96	73.43	73.63	78.49	15.04	32958	48066
	H2	80.96	74.91	70.23	76.07	70.55	69.92	73.63	18.18	52943	67121

Supplementary Table 4: Evaluation of various model performances expressed in mean percentage on the entire holdout set (including multiple ECGs for holdout patients). AUPRC: area under the precision-recall curve; AUROC: area under the receiver operating curve; DL: deep learning; ECG: electrocardiogram; XGB: XGBoost.

Time-point	Features	Model	AUROC	AUPRC	F1 score	Specificity	Recall	Precision	Accuracy	Brier Score
**30-days**	Age, Sex	XGB	65.83	2.36	3.57	57.98	64.22	1.84	58.06	1.59
	ECG	DL	82.53	28.06	27.57	71.90	77.96	16.75	72.31	5.53
		XGB	75.90	17.32	22.44	64.51	74.50	13.21	65.19	5.99
	ECG, Age, Sex	DL	83.36	29.18	28.75	73.72	77.61	17.64	73.99	5.51
		XGB	78.35	20.20	24.34	68.26	74.52	14.55	68.69	5.87
**1-year**	Age, Sex	XGB	68.31	21.90	28.04	58.56	67.56	17.69	59.60	10.42
	ECG	DL	79.01	44.35	46.68	68.00	75.56	33.77	69.34	12.25
		XGB	72.23	33.67	41.00	62.81	70.19	28.96	64.12	13.42
	ECG, Age, Sex	DL	80.20	47.08	47.96	69.80	75.65	35.11	70.84	11.91
		XGB	75.46	38.12	43.56	64.80	73.24	31.00	66.30	13.01
**5-years**	Age, Sex	XGB	73.48	59.76	58.27	66.46	67.77	51.11	66.91	20.54
	ECG	DL	77.27	65.79	64.64	68.57	72.43	58.35	70.03	19.77
		XGB	72.20	57.52	60.92	62.48	70.83	53.44	65.64	20.85
	ECG, Age, Sex	DL	79.83	69.07	66.94	71.09	74.23	60.95	72.28	19.03
		XGB	77.98	66.39	65.35	68.02	74.07	58.47	70.31	18.78

Supplementary Table 5: Full forms of ECG measurement names

**Variable**	**Definition**	**Unit**	**Short version**
Atrialrate	Atrial rate	Bpm: beats per minute	Atrial Rate
Pdur	P wave duration	Milliseconds	P duration
RRint	RR interval	Milliseconds	RR Interval
Qonset	Q wave onset		Q onset
QTcf	Fridericia Rate-Corrected QT interval	Milliseconds	Fridericia QTc
Heartrate	Heart Rate	Milliseconds	HR (or alternatively, Heart Rate)
PRint	PR interval	Milliseconds	PR interval
QRSdur	QRS duration	Milliseconds	QRS duration
QTint	QT interval	Milliseconds	QT interval
QTcb	Bazett’s Rate-Corrected QT interval	Milliseconds	Bazett’s QTc
Pfrontaxis	Frontal P axis	Degrees	Frontal P
i40frontaxis	Frontal QRS axis in Initial 40 ms	Degrees	Frontal i40msQRS
t40frontaxis	Frontal QRS axis in Terminal 40 ms	Degrees	Frontal t40msQRS
Qrsfrontaxis	Frontal QRS axis	Degrees	Frontal QRS
Stfrontaxis	Frontal ST wave axis	Degrees	Frontal ST
Tfrontaxis	Frontal T axis	Degrees	Frontal T
Phorizaxis	Horizontal P axis	Degrees	Horizontal P
i40horizaxis	Horizontal QRS axis in Initial 40 ms	Degrees	Horizontal i40msQRS
t40horizaxis	Horizontal QRS axis in Terminal 40 ms	Degrees	Horizontal t40msQRS

Qrshorizaxis	Horizontal QRS axis	Degrees	Horizontal QRS
Sthorizaxis	Horizontal ST wave axis	Degrees	Horizontal ST
Thorizaxis	Horizontal T axis	Degrees	Horizontal T
tonset	T wave onset		T onset

Supplementary Table 6: Sample sizes used for the modeling of each time point in overall data and in the experimental splits for main and lab subset. ECG: electrocardiogram; N: number.

	**Full Data**	**Development set**	**Holdout set**	**Random ECG per patient in holdout set**<br>**30-days | 1-year | 5-year**
For models with ECG only or ECG, Age, Sex features				
ECGs(n)	1605268	964741	640527	97144 | 89379 | 55650
Patients(n)	244077	146446	97631	97144 | 89379 | 55650
Episodes(n)	748773	451000	297773	97144 | 89379 | 55650
For models with Lab features (Lab results available on day of ECG acquisition)				
ECGs(n)	601307	361585	239722	56059 | 49748 | 21796
Patients(n)	141017	84564	56453	56059 | 49748 | 21796
Episodes(n)	330637	199313	131324	56059 | 49748 | 21796

Supplementary Table 7: ICD 10 codes used for the identifying diagnostic subgroups.

**Diagnoses**	**ICD Codes**
Non-ST elevation myocardial infarction (NSTEMI)	I214
ST elevation myocardial infarction (STEMI)	I210, I211, I212, I213
Heart Failure	I50, I43, I099, I110, I130, I132, I255,I420,I425,I426,I427,I428,I429,P290
Atrial Fibrillation	I48
Diabetes Mellitus	E10, E11, E12, E13, E14
Hypertension	I10, I11, I12, I13, I15
